# Management of Large Cell Neuroendocrine Carcinoma

**DOI:** 10.3389/fonc.2021.653162

**Published:** 2021-08-27

**Authors:** Virginia Corbett, Susanne Arnold, Lowell Anthony, Aman Chauhan

**Affiliations:** ^1^Department of Internal Medicine, Icahn School of Medicine at Mount Sinai, New York, NY, United States; ^2^Division of Medical Oncology, Department of Internal Medicine, Markey Cancer Center, University of Kentucky, Lexington, KY, United States

**Keywords:** LCNEC, large cell neuroendocrine carcinoma, high grade neuroendocrine carcinoma, clinical management, future directions

## Abstract

**Background:**

Large cell neuroendocrine carcinoma (LCNEC) is a rare, aggressive cancer with a dismal prognosis. The majority of cases occur in the lung and the gastrointestinal tract; however, it can occur throughout the body. Recently advances in the understanding of the molecular underpinnings of this disease have paved the way for additional novel promising therapies. This review will discuss the current best evidence for management of LCNEC and new directions in the classification and treatment of this rare disease.

**Methods:**

We performed a PubMed search for “Large cell neuroendocrine carcinoma” and “High grade neuroendocrine carcinoma.” All titles were screened for relevance to the management of LCNEC. Papers were included based on relevance to the management of LCNEC.

**Results:**

Papers were included reviewing both pulmonary and extra pulmonary LCNEC. We summarized the data driven best practices for the management of both early and advanced stage LCNEC. We describe emerging therapies with promising potential.

**Discussion:**

LCNEC are rare and aggressive neoplasms. In advanced disease, the historical regimen of platinum based therapy in combination with etoposide or irinotecan remains among the commonly used first line therapies, however for extra thoracic LCNEC regimens like FOLFOX, FOLFOIRI and CAPTEM can also be used. Further effective and safe treatment options are desperately needed. Recently, new advances including a new understanding of the genetic subcategories of LCNEC and immunotherapy agents may guide further treatments.

## Introduction

**Figure 1:**
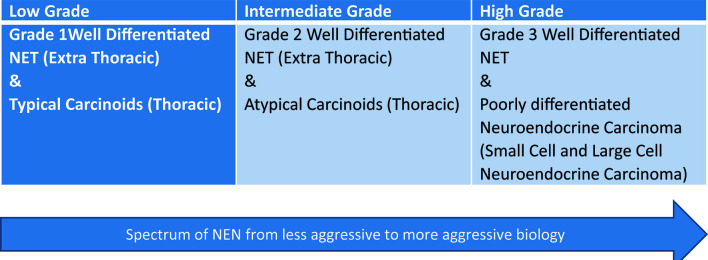
Spectrum of neuroendocrine neoplasms (NEN). This graphic demonstrates the spectrum of neuroendocrine tumors from low grade to high grade.

**Figure 2:**
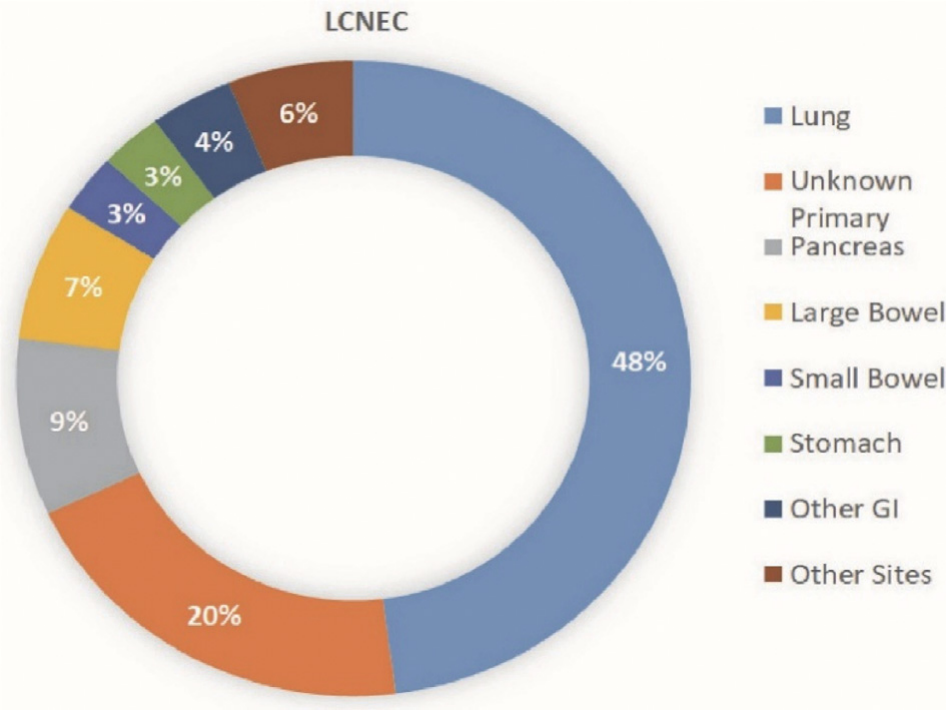
Distribution of LCNEC based on site of origin. This graphic shows the distribution of large cell neuroendocrine primary tumor locations.

Large cell neuroendocrine carcinoma (LCNEC) is a rare subgroup of high grade neuroendocrine cancer that can occur throughout the body (**Figure 1**). The most common primary site is the lung, however, LCNEC also occurs in the gastrointestinal tract and in other locations including cervix, uterus, kidney, bladder, prostate, pharynx, larynx and many other primary sites (**Figure 2**) (1–6). LCNEC is an aggressive, fast growing neuroendocrine carcinoma, similar to small cell lung cancer (SCLC). The incidence of LCNEC is increasing worldwide. Most LCNEC originates in the lungs and pulmonary LCNEC represents 2 to 3.5% of all lung cancers (7). In studies using SEER data, age adjusted incidence of pulmonary LCNEC is 0.3 per 100,000 with a rise by 0.011 people per 100,000 per year from 2004-2015 (8, 9). In addition to pulmonary LCNEC, there is evidence that LCNEC of all sites are increasing. A Dutch registry study of 47,800 patients with neuroendocrine tumors examined LCNECs, revealing an increase in incidence of LCNEC of all sites from 0.01 per 100,000 people to 1.8 per 100,000 people in 2010 (10). In this same population 5 year overall survival (OS) of patients with LCNEC was 20% (10, 11). This manuscript is intended to review current best evidence of management of this rare and deadly disease as well as several promising new directions for classification and treatment.

## Methods

The papers included in this review were primarily selected based on expert opinion. However, for completeness a PubMed search was performed for “Large cell neuroendocrine carcinoma” and “High grade neuroendocrine carcinoma.” 3658 results were obtained. All titles were screened for relevance to the management of LCNEC. Of these 606 were further examined with review of abstract and/or paper. Papers were included based on relevance to the management of LCNEC. Data search was completed on 12/31/2020. Only papers available in English were included. Author VC completed the data review and paper selection. Randomized control trial and large retrospective reviews were prioritized, as were publications using the most recent WHO neuroendocrine grading criteria for pulmonary or digestive system neuroendocrine tumors. Given limited studies available describing pure extra-pulmonary LCNEC, the majority of extra-pulmonary studies presented included both SCNEC and LCNEC. Breakdown of the number of LCNEC patients was included when available. As the GI tract is the most common site of extra-pulmonary disease, studies describing extra-pulmonary LCNEC focused on GI primary tumors.

## Histologic Classification

As with all neuroendocrine tumors correct histopathological classification of LCNEC is critical to diagnosis and treatment planning. The classification of LCNEC has evolved rapidly in the last 30 years. Pulmonary LCNEC was first named in 1991 when Travis et al. proposed that LCNEC represented a distinct category from SCLC (12). The 2004 WHO classification included a category of non-small cell lung cancer called large cell carcinoma which encompassed LCNEC and also several other subtypes of large cell carcinomas including rhabdoid phenotype, basaloid carcinoma, and lymphoepithelioma-like carcinoma (13). During this period, there was debate over whether LCNEC should be classified as a non-small cell lung cancer (NSCLC) or as a variant of SCLC (14). The 2015 WHO classification of lung cancer **(** **Table 1** **)** created the current distinct classification of pulmonary LCNEC from the more general category of large cell carcinoma. In this classification pulmonary high grade neuroendocrine carcinoma (HGNEC) including LCNEC and SCLC have mitoses >10/2mm^2^ and the presence of necrosis (13, 15). LCNEC can exist in combination with other types of lung cancer including SCLC, adenocarcinoma, and squamous cell carcinoma. Adenocarcinoma is the most common combined component; in one retrospective study median OS was not significantly different in pure LCNEC *versus* combined adenocarcinoma and LCNEC (16). 2021 WHO classification for lung neuroendocrine neoplasm will formally introduce the combined category. This will be defined as carcinoma with at least 25% of LCNEC or SCLC component mixed with non-small cell carcinoma component. LCNEC typically do not secrete vasoactive amines like some functional low grade neuroendocrine tumors, however a variety of paraneoplastic conditions have been described with pulmonary LCNEC including ectopic ACTH production (17–20), cancer associated retinopathy (21, 22), limbic encephalitis (23), Lambert Eaton Syndrome (24, 25), and syndrome of inappropriate antidiuretic hormone (SIADH) (26).

**Table 1:** Adapted from 2015 WHO classification pulmonary neuroendocrine tumors (13, 15), With recent addition of “Combined morphology” from WHO 2021 classification.

Tumor Type	Morphology	Mitoses (Mitoses/2 mm2)	Necrosis	Other Characteristics:
Typical carcinoid	Carcinoid	<2	No necrosis	Carcinoid morphology an <2 mitoses/2mm2, lacking necrosis >0.5cm
Atypical carcinoid	Carcinoid	2-10	Necrosis	Carcinoid morphology with 2 to 10 mitoses/2mm2, or necrosis
Large cell neuroendocrine carcinoma	Neuroendocrine	>10	Necrosis	Cytological features of NSCLC.
Small cell lung cancer	Neuroendocrine	>10	Necrosis	Cytological features of SCLC
Combined with NSCC component	Up to 25% LCNEC or 25% Small cell component in combination with NSCC			

NSCC, Non-small cell cancer; SCLC, Small cell lung cancer.

Gastrointestinal (GI) LCNEC has also undergone a recent evolution in classification. The key distinction between well-differentiated neuroendocrine tumors and high grade neuroendocrine carcinoma was initially described in the 2000 WHO guidelines, which classified these tumors as poorly differentiated. The 2010 guidelines changed to include distinct groups including (i) grade 3 neuroendocrine carcinoma which encompassed both LCNEC and small cell neuroendocrine carcinoma (SCNEC), (ii) and all tumors with Ki-67 greater than 20 and mitotic index greater than 20 (27). The 2010 criteria also described a new category of mixed adenoneuroendocrine carcinoma (MANEC) with an adenocarcinoma and neuroendocrine component. The 2010 criteria definition of G3 as tumors with mitotic rate >20 mitosis per 2 mm2 and Ki-67 >20% included both well differentiated neuroendocrine tumors with high proliferation and poorly differentiated HGNEC such as LCNEC and SCNEC. The WHO 2019 GI NET criteria included a new category of well differentiated (G3) neuroendocrine tumors **(** **Table 2** **).** This update reflects the recognition that well differentiated tumors can be high grade but are distinct from SCNEC and LCNEC. The WHO 2019 grading system also changed the terminology of mixed tumors to mixed neuroendocrine-nonneuroendocrine neoplasm (MiNEN) (29).

**Table 2:** Adapted from 2019 WHO classification of tumors of the digestive system (28).

Terminology	Differentiation	Grade	Mitotic rate (mitoses/2 mm2)	Ki-67 (Percent)
NET, G1	Well differentiated	Low	<2	<3%
NET, G2	Well differentiated	Intermediate	2-20	3-20%
NET, G3	Well differentiated	High	>20	20%
NEC, SCNEC	Poorly differentiated	High	>20	>20%
NEC, LCNEC	Poorly differentiated	High	>20	>20%
MiNEN	Well or poorly differentiated	Variable	Variable	Variable

NET, Neuroendocrine tumor; SCNEC, Small cell neuroendocrine carcinoma; LCNEC, Large cell neuroendocrine carcinoma; MiNEN, Mixed neuroendocrine-non neuroendocrine neoplasm.

## Clinical Characteristics of Pulmonary and Extra-Pulmonary LCNEC

Pulmonary and extra-pulmonary LCNEC share many similar clinical characteristics. Patients with pulmonary LCNEC were more likely to be white males (9, 30). Patients with pulmonary LCNEC are less likely to present with advanced stage disease as compared to SCLC (31). Cough is the most common presenting symptom of pulmonary LCNEC (32). Although pulmonary LCNECs can occur throughout the lungs, they are most likely to occur in the upper lung lobes (33). In large studies of GI HGNEC, most patients are also men, most patients are in their 60s, and most are white. The majority of patients also presented with stage IV disease. In one large study the most common primary site was the colon, followed by pancreas, and esophagus (34). Other studies showed that the stomach and small intestine were also frequent sites of HGNEC (35). Pulmonary LCNEC is commonly associated with a smoking history (36, 37). In limited data available, patients with a history of colorectal HGNEC are slightly more likely to describe a history of smoking but the contrast with nonsmokers is less pronounced than in pulmonary disease. In addition common presenting symptoms include abdominal pain, hematochezia, melena, and altered bowel movements (38). Although data is limited in extra-pulmonary LCNEC and OS is variable depending on primary site (39), in large studies 5 year OS in pulmonary LCNEC was 16.7% (9) and 13.3% (40) in colorectal HGNEC. Brain metastasis are more common in pulmonary LCNEC (12-19.2% at presentation in large studies) (8, 41) *versus* 1.6% in GI HGNEC (31).

## Molecular Characteristics of LCNEC

The most recent important innovations in LCNEC classification have occurred in genomic analysis of these tumors. Prior reviews have comprehensively examined the molecular characteristics of pulmonary LCNEC (42). Briefly, George et al. performed genomic and transcriptome analysis on pulmonary LCNEC, comparing genomic alterations in LCNEC with adenocarcinomas and squamous cell carcinoma. They found frequent mutations in *TP53*, *RB1*, *STK11* and *KEAP1*. They also describe less common mutations in the RAS-pathway including *KRAS*, *NRAS*, Harvey rat sarcoma viral oncogene homolog (*HRAS*). They identified two molecularly defined subgroups. The first “type 1 LCNECs” with *STK11/KEAP1* alterations and a neuroendocrine profile similar to SCLC with elevated *ASCL1* and *DLL3*. George et al. also describe “type II LCNEC” which has a genetic resemblance to SCLC with *TP53* and *RB1* alterations, but with reduced activity of typical neuroendocrine markers and higher *NOTCH* pathway activation (43). PD-L1 (programmed death-ligand 1) and tumor mutational burden (TMB) are known to be predictors of potential response to immunotherapy (44) and have been evaluated in LCNEC. PD-L1 was evaluated in relation to the LCNEC subgroups revealing that tumor PD-L1 ≥ 1% is expressed in 16% of metastatic LCNEC and PD-L1 expression was not significantly different between the molecular subgroups of LCNEC (45). Many other studies have reported a similar percentage of PD-L1 expression in pulmonary LCNEC (**Table 3**). Reported values for PD-L1 expression in tumor cells (TC) range from 9.1% (53) to 74% (51). PD-L1 expression was more common in stromal lymphocytes or local immune cells (IC) and ranged from 37% (48) to 75% (47).

**Table 3:** Key papers describing PD-L1 expression in pulmonary LCNEC.

Study	Number of patients	PD-L1 antibody test	PD-L1 Cutoff for positivity	PD-L1 Expression Tumor Cells (TC)	PD-L1 Expression in Stromal Lymphocytes “Immune Cells” (IC)	Other findings
Abdel Karim et al. (46)	LCNEC (n=24)	LCNEC tissue microarray (TMA) from US Biomax	3/5 cases with positive TCs had 1% or less tumor staining 5/10 cases with positive ICs expressed 2% or less positive cells.	5/24 (21%)	10/24 (42%)	No correlation between Ki-67 and stage of disease.
Arpin et al. (47)	LCNEC (n=68)	Anti-PD-L1 22C3 antibody (kit and automat Dako, Dako, Agilent, USA)	Positive TC % of TCs with membrane PD-L1 labeling with >1% Positive ICs = the % of the IC surface labeled, IC negative = <1%; IC1 = 1–5%; IC2 = 5–10%; and IC3 = >10%	7/68 (11%)	49/65 (75%) (with >1% expression)	20/65 IC with >10% PD-L1. Median overall survival was significantly shorter for metastatic LCNEC patients with TC +/IC- samples as compared to TC+/IC- samples.
	• 65 assessable for IC score• 68 assessable for TC score					
Eichhorn et al. (48)	LCNEC (N=76)	Antibody Clone: SP263; Ventana Benchmark Ultra, Ventana Medical Systems, AZ 85755, USA	>1%	17/76 (22%)	28/76 (37%)	Patients with both positive TC and negative IC had significantly worse 5 year survival.
Guleria et al. (49)	LCNEC (N=11)	Antibody clone SP263, (VENTANA Medical Systems, Inc)	>1% positive tumor cells	4/11 (36%)	5/11 (45%)	Variable PD-L1 expression on literature review.
Hermans et al (45)	LCNEC (N=98)	Monoclonal rabbit anti-PD-L1 clone 28-8 DAKO Autostainer Link 48 system with the PD-L1 IHC 28-8 pharmDx kit (DAKO, Agilent, USA)	Tumor proportion score (TPS) defined as % of tumor cells with complete or partial membranous staining at any intensity. TPS ≥ 1% was considered as positive.	16/98 (16%)	N/A	CD8 expression also documented. CD8 expression in tumor and stroma correlated with PD-L1 expression and improved overall survival.
Inamura et al. (50)	LCNEC (n=41)	Anti‐PD‐L1 rabbit monoclonal antibody (clone: E1L3N; Cell Signaling Technology, Danvers, MA; diluted 1:50)	≥5% were categorized as “PD‐L1 positive”	11/41 (27%)	N/A	In combined assessment of 74 SCLC and 41 LCNEC PD-L1 expression was significantly associated with lower overall mortality.
Kasajima et al. (51)	LCNEC (N=58)	Mouse monoclonal antibody directed against PD-L1 (clone 22C3, dilution 1:30, Dako, Glostrup, Denmark)	≥1% was considered as PD-L1 positive	5/58 (9%)	25/58 (44%)	In combined assessment of 127 SCLC and 58 LCNEC samples, PD-L1 positivity in IC but not TC was associated with CD8+ infiltration, increased tumor associated inflammation, and improved overall survival.
Kim et al. (52)	LCNEC (N=72)	Human B7-H1/PD-L1 antibody (R&D Systems, Minneapolis, MN)	3 categories of PD-L1 positivity reported	(>1%) 12/72 (17%)	(>1%) 33/72 (46%)	IC infiltration and PD-L1 expression on IC was more strongly correlated in LCNEC as compared to SCLC.
			TC1/IC1 1%- 9%			
			TC2/IC2 10%-49%			
			TC3/IC3 50%+			
Ohtaki al (53).	LCNEC (N=95)	Rabbit monoclonal antibodies against PD-L1 (Cell Signaling, E1L3NR, 1:200 dilution)	Tumors with score ≥ 1 were graded as PD-L1 positive	70/95 (74%)	N/A	Patients with PD-L1 positive TC had better but not significantly better recurrence free survival.
Tsuruoka et al. (54)	LCNEC (N=106)	Rabbit monoclonal antibody (E1L3N, 1:800, Cell Signaling Technology, Inc., Danvers, MA, USA)	Semi quantitative H-score method, score used by multiplying the percentage of tumor area by staining intensity. Score of 1 was used as cutoff.	11/106 (10.4%)	N/A	PD-L1 status was also assessed in SCLC and was 5.8% as compared to 10.4% in LCNEC.

PD-L1, Programmed death-ligand 1; SCNEC, Small cell neuroendocrine carcinoma; LCNEC, Large cell neuroendocrine carcinoma; N/A, not available.

Further investigations into molecular subtypes of pulmonary LCNEC will have important treatment implications. Rekhtman et al. explored next generation sequencing in pulmonary LCNEC. This study revealed three clusters, each with similar molecular make up, including 40% of cases with a “SCLC- like” with *TP53* and *RB1* alterations, and 56% of cases described as “NSCLC- like” with *STK11*, *KRAS*, *KEAP1-NFE2L2* alterations which are more typical of adenocarcinoma or squamous cell carcinoma. This study also showed both of these groups had elevated TMB slightly above 10 mutations/Megabase (Mb). Interestingly, in 4% of patients they identify a “carcinoid-like subset” with MEN1 alterations and low mutational burden (55). Another group also examined mutational burden in pulmonary HGNEC including 39 LCNEC and 63 SCLC cases. They found that the number of non-synoumous mutations was significantly increased in patients with IC infiltration and PD-L1 expression on ICs but not correlated to PD-L1 expression on TCs (52). Another study examined TMB in pulmonary HGNEC in 300 patients with LCNEC and 887 with SCLC. They found the median TMB in patients with pulmonary LCNEC was 9.9 mutations/Mb, the same as in the SCLC group (56).

The overlap between LCNEC and atypical carcinoid tumors was also explored in another study. One group used clustering analysis to compare the genomic profiles of 116 pulmonary carcinoids (including 35 atypical carcinoids), 75 LCNEC and 66 SCLC. Of note, 6 atypical carcinoids clustered with LCNEC patients with a similar survival. The authors suggest that these “supra carcinoids” may represent a group of atypical carcinoid tumors that progress to LCNEC over time (57).

One study examined driver gene status in 94 LCNECs. In this study EGFR mutants were detected from peripheral blood with an amplification refractory mutation system (ARMS) and anaplastic lymphoma kinase (*ALK*) rearrangement was detected by immunohistochemistry. They found driver mutations including epidermal growth factor receptor (*EGF*R) (8.3%), anaplastic lymphoma kinase (*ALK*) (5.8%) (58). Given the limited number of cases the percentage of patients with *ALK*-mutated LCNEC that respond to tyrosine kinase inhibitor (TKI) treatment is not known. Limited case reports exist of patients with pure and mixed LCNEC who have *ALK* rearrangements, and a few patients experienced benefit from treatment with ALK inhibitors (59, 60). In another study primary tumor location in 125 case of pulmonary LCNEC was examined. Most patients had peripheral tumors. Central tumors were associated with smoking history. Genomic DNA was used for polymerase chain reaction (PCR) amplification and sequencing of *EGFR*. *RB1* was detected on IHC of tumor sample. Peripheral tumors had a higher incidence of *EGFR* expression. RB1 protein was more frequently expressed in peripheral tumors. In a multivariate analyses of tumor location, resection status, and *EGFR* mutational status were associated with improved OS (61). Additional case reports of *EGFR* mutations in LCNEC have been described, along with response to gefintib (62). These findings suggest a potential role for molecular profiling in LCNEC.

There is less information available about the molecular underpinnings of gastrointestinal LCNEC. A retrospective analysis of pancreatic neuroendocrine carcinoma included 49 patients with SCNEC and 18 patients with LCNEC. Immunohistochemistry was reviewed and pRb proteins were identified by monoclonal antibodies. *KRAS* codon 12 mutations were evaluated using fluorescence-based DNA analysis, and the PCR. Of the LCNEC patients, 17 were evaluable for genetic analysis and 4 patients, (23.5%), had retained *RB* and *KRAS* (wild type) and 14 (82.3%) had *RB* loss and/or *KRAS* mutation. Of the 18 patients with LCNEC of the pancreas the response rate to first line platinum-based therapy was 40% compared to 65.2% in the 31 SCNEC patients. They analyzed the combined pancreatic LCNEC and SCNEC cohort and found that *RB* and *KRAS* mutations were associated with improved response to platinum-containing therapy (63).

Another study examined next generation sequencing and pathology of 19 poorly differentiated pancreatic neuroendocrine carcinomas with 10 LCNEC and 9 SCNEC. Immunohistochemical labeling was done for proteins including pRb, p53, PAX8 among others. Genomic DNA was extracted from and PCR amplification was performed on genomic DNA or whole-genome amplification for *KRAS*, *TP53*, *CDKN2A/p16*, and *RB1*. In this study 5 of 10 LCNEC patients expressed PDX1 (pancreatic duodenal homeobox 1), 5 of 10 LCNEC also expressed PAX, a transcription factor expressed in well differentiated pancreatic neuroendocrine tumors. Abnormal *TP53* expression was found in 9 of the 10 LCNEC cases and *RB1* expression loss in 6 of 10 LCNEC patients. Of note, overexpression of *BCL-2* was observed in 9 of 9 of the SCNECs and 5 of the 10 LCNECs (64).

Although testing for microsatellite instability (MSI) is routinely done for colorectal adenocarcinoma, few studies reported testing for MSI in gastrointestinal neuroendocrine carcinoma. One study reviewed 40 cases of gastrointestinal neuroendocrine carcinoma including both SCNEC and LCNEC, with 10 cases of colorectal neuroendocrine carcinoma (5 SCLC and 5 LCNEC). MSI cases were detected using immunohistochemistry for the mismatch repair (MMR) proteins MLH1, PMS2, MSH2, and MSH6. Of the 40 cases included, MSI was found in 3 cases, all were cecal neuroendocrine carcinomas (65). Limited studies have evaluated PD-L1 expression in GI HGNEC **(** **Table 4** **).** In one study of 33 patients with GI HGNEC 29% of patients had PD-L1 expression in TCs. TMB was also measured with a range from 0.57 to 11.75 mutations/Mb, with a median TMB of 5.68 mutations/Mb (67). In gastric NEC (4 LCNEC and 39 SCNEC) 48.8% of cases in the combined cohort had TC expression of PD-L1 (68).

**Table 4:** Key papers describing PD-L1 expression in gastrointestinal HGNEC.

Study	Number of patients	PD-L1 antibody test	PD-L1 Cutoff for positivity	PD-L1 Expression Tumor Cells (TC)	PD-L1 Expression in Stromal Lymphocytes “Immune Cells” (IC)	Other findings
Roberts et al. (66)	GI LCNEC (N=25)	Clone E1L3N; Cell Signaling, Danvers, MA; dilution: 1:200	For TCs, positive expression >1%	3/25 (10%)	5/25 (20%)	In assessment of combined 12 SCNEC and 25 LCNEC PD-L1 was positive on 14% of TCs and 27% of ICs.
			For ICs positive expression ≥1			
Xing et al. (67)	GI HGNEC (SCLC/LCNEC not reported) (N=33)	Clone 22C3, dilution 1:50; M365329-8CN, Dako	>1%	9/31 (29%) of HGNEC of the GI tract	N/A	TMB was also measured, range 0.57 to 11.75 mutations/Mb, with a median of 5.68 mutations/Mb
Yang et al. (68)	Gastric HGNEC (N=43) LCNEC = 4	PD-L1 (1:500, ab205921, Abcam)	PD-L1 expression score. Positive cell score x staining intensity. Final score ≥ 4 were classified as PD-L1 high.	21/43 (48.8%) cases (combined LCNEC and SCNEC)	N/A	PD-L1 positive patients had significantly shorter overall survival.
	SCNEC = 39					

PD-L1, Programmed death-ligand 1; SCNEC, Small cell neuroendocrine carcinoma; LCNEC, Large cell neuroendocrine carcinoma; HGNEC, High grade neuroendocrine carcinoma; TMB, Tumor mutational burden; Mb, Megabase; N/A, not available.

As previously described studies in pulmonary LCENC have suggested that a possible subset of atypical carcinoid tumors progress to LCNEC over time (57). A similar study was done in gastroenteropancreatic tumors which evaluated a next generation sequencing analysis of neuroendocrine neoplasms with unsupervised cluster analysis which revealed 3 histology independent clusters. Each cluster contained similar genetic mutations shared by neuroendocrine tumors (NETs) and HGNEC, including well differentiated NETs with high grade components. The authors suggest that some NETs may progress to secondary LCNEC over time (69). Although these theories are still novel and require further exploration, they highlight the many recent innovations in genetic analysis of these tumors.

## Diagnosis

Biopsy must be obtained to confirm diagnosis. Diagnosis is made based on neuroendocrine morphology, mitotic count, presence of necrosis, and proliferation rate as assessed by Ki-67. Confirmation of neuroendocrine differentiation is needed using immunohistochemical markers. Core biopsy samples are preferred as in small tumor samples diagnosis can be difficult in fine needle aspirate due to crushed tumor cells (15). It is to be noted that LCNEC are often diagnosed postoperatively based on surgical specimen. Limited fine needle aspirate tissue especially from lung lesion is often not able to confirm the diagnosis. Despite the preference for larger samples endobronchial ultrasound-guided transbronchial needle aspiration has been shown to be effective in diagnosis of LCNEC (70). For pulmonary and gastrointestinal primary sites cross sectional imaging should be done for staging including CT of the chest, abdomen, and pelvis with intravenous contrast. If a gastrointestinal LCNEC primary is suspected, further imaging with MRI of the abdomen and pelvis with contrast (Eovist preferred) while non-contrast chest CT can be considered. MRI of the brain should also be considered for staging at presentation in pulmonary LCNEC. A small retrospective study of 37 patients with LCNEC compared to 76 patients with SCLC, revealed 16.2% of LCNEC patients presented with brain metastasis compared to 18.5% in SCLC patients (71). FDG- PET/CT is also recommended at baseline for assessment of disease burden. Somatostatin receptor scintigraphy with imaging such as Gallium-68 DOTATATE PET/CT can be considered in select cases, especially if the clinical course is following a relatively indolent course, for potential screening for somatostatin receptor type 2 (SSTR-2) targeted clinical trials. One large retrospective study of gastroenteropancreatic neuroendocrine neoplasms examined SSTR-2 in 163 patients with high grade gastroenteropancreatic neuroendocrine tumors or cancer of unknown primary. Of this group 128 were patients with LCNEC, with 36 patients (22.1%) strongly positive for SSTR-2 on immunohistochemical examination (72). Another study examined SSTR-2 expression retrospectively in 218 pulmonary neuroendocrine tumors, including 60 cases of LCNEC. In this study 20 (33%) of these patients were positive for SSTR-2 on immunohistochemical examination (73).

Unlike well-differentiated neuroendocrine tumors, high grade neuroendocrine carcinomas are rarely functional. Some institutions use peripheral blood measurements of chromogranin A (CgA) and neuron specific enolase (NSE) as these have been shown to be elevated in some patients with high grade neuroendocrine carcinoma including SCLC and some LCNEC patients (74). In SCLC elevated NSE is a negative prognostic factor (75). NSE was also examined in a retrospective cohort of poorly differentiated gastroenteropancreatic neuroendocrine tumors, which included both LCNEC and SCLC. In this combined cohort elevated NSE greater than two times the upper limited of normal was associated with a worse overall survival (76).

As described above emerging data suggests a potential role for next generation sequencing in patients with LCNEC of all primary sites. Although there is not yet robust data to suggest testing in all patients, next generation sequencing should be considered.

## Treatment

### Stage I-III

Surgery should be considered first line treatment in all patients with early stage pulmonary (I-III) LCNEC. Retrospective data suggests that in pathologic limited stage disease, surgical resection, most commonly lobectomy, was associated with a 5 year OS of 49.2% (77). In a large propensity-matched retrospective study using SEER data in patients with pulmonary LCNEC, surgery was associated with improved overall survival. This study examined 473 patients with pathologic stage IA pulmonary LCNEC and compared outcomes with patients with lung adenocarcinoma and squamous cell cancer. For LCNEC patients median OS was 66.0 months and 5 year OS rates 52.5% (33). In terms of the type of surgery, for pathologic stage I and II pulmonary LCNEC, lobectomy and pneumonectomy have been shown to have improved survival (78). To better evaluate the optimal type of surgery Lufti et al. retrospectively examined patients with pathologic stage I LCNEC and compared 5 year OS in patients who had a sub-lobar resection (wedge or segmentectomy) as compared to patients who had lobectomy. They found sub-lobar resection in early stage LCNEC was associated with a lower 5-year OS rate compared to lobectomy on unadjusted and propensity matched analyses (79).

A retrospective study of 139 patients with pulmonary LCNEC was done, all patients underwent surgery (majority were lobectomies) with curative intent. In this group 5 year OS was 53%, 5 year disease free survival was 39% (80). Although there is less data available in extra pulmonary LCNEC, limited studies of high grade gastroenteropancreatic neuroendocrine carcinoma show improved survival in patients who undergo resection for early clinical stage disease. One retrospective study reviewed 600 high grade high-grade gastroenteropancreatic neuroendocrine tumors with 335 patient who presented with limited clinical stage at diagnosis. Of these patients 89% underwent surgery and median survival for patients undergoing surgery was 153 months *vs.* 71 months for those not undergoing surgery (81). Another study reviewed outcomes in high grade pancreatic neuroendocrine carcinoma patients undergoing surgery. This group included 28 patients who underwent surgery and 14 patients who underwent resection of the primary tumor in clinically early stage disease with curative intent. All patients recurred, the median time to recurrence or metastasis in this group was 7 months (range 2–14 months). Resection of the primary tumor was an independent prognostic factor of improved survival for patients after occurrence of metastatic disease (82).

Three prospective clinical trials have evaluated adjuvant treatments for pulmonary LCNEC (**Table 5**). They include a one arm nonrandomized trial of adjuvant cisplatin and etoposide, compared to a control of historical data from the same institution. In this study 15 men received adjuvant cisplatin and etoposide for 1 or 2 cycles after completing lobectomy with lymph node dissection for LCNEC. This trial revealed a 5 year OS of 88.9% as compare to historical control of 47.4% (83). Another prospective phase II study evaluated 4 cycles of adjuvant irinotecan and cisplatin after complete resection of pulmonary LCNEC and SCLC tumors (95% of patients received a lobectomy). In the 23 patients with LCNEC, OS at 3 years was 86% (84). Recently the results were published of a phase III randomized control trial comparing adjuvant cisplatin and irinotecan *versus* etoposide plus cisplatin in patients with pathologic stage I-IIIA resected HGNEC. The initial primary endpoint of this study was overall survival; however, the primary endpoint was changed to relapsed free survival (RFS) as there were too few events for analysis of OS after accrual was completed. In total 221 patients were enrolled, of these 74 had pure LCNEC (38 were treated with etoposide and cisplatin and 36 with irinotecan and cisplatin). There was no significant difference in 2 year RFS between the treatment arms, etoposide and cisplatin 65.4% *vs* irinotecan and cisplatin 69%, P = 0.619. Median RFS was not reached. In a subgroup analysis there was no significance difference among patients with LCNEC or combined LCNEC (85, 93).

**Table 5:** Key Papers Describing Adjuvant Therapy in Pulmonary LCNEC.

Study	Study Design	Patient population	Total Patients	Treatment	Outcomes	Other
Iyoda et al. (83)	Prospective, one arm nonrandomized clinical study, compared to historical control group.	LCNEC after surgical resection (majority of patients underwent lobectomy)	15 LCNEC	Etoposide / Cisplatin	2 year OS 88.9% in the adjuvant chemotherapy group *vs* 65.2% in the control group. 5 year OS 88.9% in adjuvant chemotherapy group *versus* 47.4% in control group.	
Kenmotsu et al. (84)	Prospective, one arm phase II trial	Stage I-IIIA completely resected pulmonary HGNEC (both SCLC and LCNEC)	40 (23 LCNEC and 17 SCLC)	Irinotecan / Cisplatin	3 year OS 86% among 23 LCNEC patients and 75% among 17 SCLC patients.	
Kenmotsu et al. (85)	Prospective randomized, open-label, phase III study	Stage I-IIIA completely resected pulmonary HGNEC (both SCLC and LCNEC)	221 (74 LCNEC and 78 SCLC, 39 combined SCLC and 20 combined LCNEC)	Etoposide/Cisplatin (111 patients) *vs* Irinotecan / Cisplatin (110 patients)	No significance difference in 3 year RFS between treatments arms (Etoposide/Cisplatin arm 65.4% and Irinotecan/Cisplatin arm was 69%). Also no significant difference between treatment arms in subgroups of LCNEC and SCLC.	
Rossi et al. (86)	Retrospective review	Pure pulmonary LCNEC who underwent surgical resection	83	Variable	5 year OS 27.6%, stage I, 33%, stage II, 23% and stage III, 8%. Of 13 patients who received adjuvant chemotherapy patients with SCLC based treatment (cisplatin/etoposide) had improved overall survival compared to other NSCLC based treatments.	
Veronesi et al. (87)	Retrospective review	Consecutive patients with surgical resection of pulmonary LCNEC	144	Variable	5 year OS was 42.5%; for stage I, 52%, stage II, 59%, and stage III, 20%. Patients with stage I disease who received chemotherapy tended to survive longer than those who received no chemotherapy (p = 0.077).	
Iyoda et al. (88)	Retrospective review	LCNEC patients with surgical resection of primary tumors	72	Variable	5 year DFS was 42.7%, 5 year OS for patients with recurrent tumors was 12.5%. Receiving platinum based adjuvant chemotherapy was associated with lower rate of tumor recurrence as compared to non-platinum based adjuvant chemotherapy.	
Sarkaria et al. (89)	Retrospective Review	Resected LCNEC	100	Variable	Median OS was 3.4 years. 20 of 71 patients with stage I-II resected disease received platinum based chemotherapy. The 5 year OS was 37% for patients who did not receive platinum based chemotherapy and 51% for patients who received it.	
Saji et al. (90)	Retrospective Review	Resected LCNEC or mixed LCNEC	45	Variable	2 year OS was 89.2% and 5 year OS was 69.4%. 5 year survival rates of patients who underwent perioperative adjuvant chemotherapy was significantly higher (87.5%) than those who underwent surgery alone (58.8%).	
Kujtan et al. (91)	Retrospective review	Stage I LCNEC	1232 (957 surgical resection alone, 275 both surgery and systemic chemotherapy)	Variable	OS in univariate 30 day analysis was significantly improved in patients who received chemotherapy across the whole cohort. 5 year OS was significantly improved in patients who received both surgery and systemic chemotherapy as compared to those who received chemotherapy alone (64.5% *versus* 48.4%).	
Raman et al. (92)	Retrospective review	Stage I LCNEC	2642	Variable	5 year OS was 53%, univariable analysis showed a significant increase in OS with adjuvant therapy for stage I LCNEC compared with no adjuvant therapy. In subgroup analysis of stage IA patients (n=2055) there was no significant survival benefit for adjuvant therapy, however significant survival benefit was present in stage IB patients (N= 587).	
Shen et al. (58)	Retrospective review	Surgically resected LCNEC	94	Variable	Of 75 patients who received adjuvant chemotherapy patients who received platinum /etoposide base regimens had improved DFS as compared to NSCLC regiments.	

RFA, recurrence free survival; OS, overall survival; LCNEC, Large cell neuroendocrine carcinoma; SCLC, Small cell Lung Cancer; DFS, Disease free survival; NSCLC, Non-small cell lung cancer.

Several retrospective reviews exist of adjuvant therapy for pulmonary LCNEC. Most studies also suggest benefit of adjuvant chemotherapy after resection for early stage disease. In a large retrospective study of 1232 resected pathologic stage I pulmonary LCNEC 5 year OS was significantly improved in patients who received both surgery and systemic chemotherapy as compared to those who received chemotherapy alone (64.5% *versus* 48.4%) (91). Saji et al., retrospectively reviewed 45 patients with pulmonary LCNEC who underwent surgical resection and received perioperative chemotherapy (7 with induction and 16 with adjuvant therapy). They found significantly improved 5 year OS in patients who received perioperative chemotherapy (87.5%) compared to those who received surgery alone (58.8%) (90). A large retrospective study of 1232 patients who underwent surgical resection for pathologic stage 1 LCNEC included 957 patients with surgery resection alone and 275 who received surgery and chemotherapy. The majority of patients in this study had stage IA disease. 5 year OS was significantly improved in patients who received chemotherapy (64.5%) as compared to patients who received surgery alone (48.4%) (91). In another large retrospective study subgroup analysis was done for stage IA *versus* stage IB cases. They found in subgroup analysis of stage IA patients (N=2055) there was no significant survival benefit for adjuvant therapy, however significant survival benefit was present in stage IB patients (N= 587) (92).

In summary most studies suggest that adjuvant chemotherapy improves OS in patients with localized LCNEC. The evidence for this is best in stage IA disease. Platinum based chemotherapy with either irinotecan or etoposide is commonly used. The best evidence for these two agents is the prospective, randomized phase III trial from Kenmotsu et al. (85) which found no significant difference in 3 year RFS between these two regimens.

In extra pulmonary LCNEC no prospective studies were identified evaluating adjuvant chemotherapy. Several retrospective studies have been done **(** **Table 6** **)**, most include HGNEC with cohorts combining patients with SCNEC and LCNEC. In a review of 119 patients with high grade pancreatic neuroendocrine carcinoma patients who underwent surgical resection there was improved 3 year OS compared to those without resection. The patients who underwent resection in this study also underwent adjuvant chemotherapy, the majority with cisplatin and etoposide or carboplatin and etoposide. These data suggests that patients who could tolerate more than four courses of adjuvant chemotherapy had improved outcomes (82). In another retrospective study of 39 gastric high grade neuroendocrine carcinomas, including 39 SCNECs and 4 LCNECs, all patients had surgical resection and most received postoperative chemotherapy. The largest group (14) of patients received adjuvant fluoropyrimidine-based regimens, including 5-fluorouracil, leucovorin, oxaliplatin combination regimen (FOLFOX) and capecitabine plus oxaliplatin. For those that received postoperative chemotherapy the median overall survival was 44 months compared to 15 months in 5 patients who did not receive chemotherapy post-operatively (96). In a series of 126 patients with colon, rectal, and anal high grade neuroendocrine carcinoma, surgical resection and adjuvant chemotherapy in localized disease did not improve overall survivial (94). A recent large study of 759 patients in the National Cancer Database examined outcomes with post-operative chemotherapy after curative resection. In this study 37.7% of patients had primary tumors in the pancreas, 25.7% had gastric, and 36.6% of patients had tumors that originated in the small intestine. This study used inverse probability of treatment weighting (IPTW) to reduce selection bias and compared post-operative chemotherapy with observation. In this study, 28.1% of patients received postoperative chemotherapy after curative resection, and IPTW showed no OS benefit in the overall group. In a subgroup analysis, paradoxically there was improved survival benefit in the observational arm of the small intestinal group as compared the postoperative chemotherapy group (35). Taken in total the data for post-operative chemotherapy after surgery is mixed. One explanation for mixed results is that the very aggressive nature of this disease leads to recurrence of disease despite surgical resection and adjuvant chemotherapy. Further trials, especially prospective, randomized control trials are needed to clarify the true benefit of adjuvant chemotherapy in resected GI HGNEC.

**Table 6:** Adjuvant therapy extra-pulmonary LCNEC (most studies report outcomes for combined SCNEC and LCNEC patient populations).

Study	Study Design	Patient population	Total Patients	Treatment	Outcomes	Other
Smith et al. (94)	Retrospective review	HGNEC of the colon and rectum	126 HGNEC	Variable	3 year OS was 8.7%, median survival 13.2 months 5 year OS 5% in metastatic disease and 18% in non-metastatic disease Of 7 patients with HGNEC 5 received adjuvant chemotherapy	
Haugvik el al. (95)	Retrospective review	High grade pancreatic neuroendocrine carcinoma	119 patients	Variable	3 year survival rate after primary surgery and metastatic disease was 69%. Patients who underwent combined surgery and chemotherapy had significantly improved survival as compared to patients who received chemotherapy alone.	
Liu et al. (96)	Retrospective review	High grade gastric neuroendocrine carcinoma	43 (39 SCNEC and 4 LCNEC) all underwent surgical resection (5 palliative resections)	Variable	3 year OS was 44.51%, 5 year OS was 35.05%. 34 patients had adjuvant chemotherapy with median OS 44 months as compared to 14 months in 5 patients who did not receive post-operative chemotherapy.	
Fields et al. (40)	Retrospective review	High grade neuroendocrine carcinoma of the colon and rectum.	1208 patients (653 SCNEC and 556 LCNEC)	Variable	Median OS 9.0 months, 3 year OS was 17.8% and 5 year OS was 13.3%. For localized disease 5 year OS was 15.9% in patients who received only chemotherapy, 31.7% only surgery, and 37% for surgery and chemotherapy.	
Alese et al. (34)	Retrospective review	High grade neuroendocrine carcinoma of the gastrointestinal tract	1861 patients	Variable	519 patients underwent surgery, 224 patients received post-operative chemotherapy which was associated with improved OS as compared to patients that did not receive post-operative chemotherapy.	
Schmitz et al. (35)	Retrospective review	High grade neuroendocrine carcinoma of the stomach, small bowel, or pancreas	759 patients	Variable	213 patients received post-operative chemotherapy after curative resection. For these patients post-operative chemotherapy was not associated with improved overall survival. 5 year OS in the chemotherapy group was 39% compared to 45% in patients that did not receive post-operative treatment	

HGNEC, High grade neuroendocrine carcinoma; OS, overall survival; LCNEC, Large cell neuroendocrine carcinoma; SCNEC, Small cell neuroendocrine carcinoma.

### Radiation

In several retrospective studies post-operative radiation after resection for early stage pulmonary LCNEC does not improve survival. In addition when compared with surgery followed by radiation, patients who received surgery combined with chemotherapy had improved survival (97, 98).

In patients with early stage pulmonary LCNEC that are not surgical candidates, radiation may be considered as primary therapy. For patients with stage III disease (not surgical candidates) radiotherapy was associated with a significant increase in overall survival (97). One retrospective study of SEER data used propensity-matched analysis to compare stereotactic ablative body radiotherapy (SABR) to conventionally fractionated radiation therapy in patients with early stage pulmonary LCNEC that were not surgical candidates. In this study SABR was associated with improved OS (99).

There is limited data on the role of prophylactic cranial radiation (PCI) in early stage LCNEC. In retrospective studies PCI is used significantly less in LCNEC as compared to SCLC, and in one study, only 4% of patients received PCI (100). Cumulative risk of brain metastases in LCNEC increases over time in patients with advanced disease treated with first line carboplatin and etoposide. In patients with stage III or IV disease at diagnosis, the risk of brain recurrence reaches 58% to 48% at 18 months after diagnosis (71). Another large study found that in 23 patients with metastatic LCNEC without brain metastasis, 6 (23%) went on to develop brain metastases during clinical follow up (41). Another study of pulmonary LCNEC using SEER data noted a rate of brain metastases of 19.2% as compared to 16.7% in SCLC (8). A retrospective study of 72 patients with advanced or metastatic LCNEC and SCLC included 21 patients treated with PCI (17 SCLC and 4 LCNEC). In this small group of LCNEC patients, PCI was associated with higher progression free survival (PFS) and median OS (101). In another retrospective study, 70 patients with pulmonary LCNEC were reviewed. Almost all (94%) of patients underwent surgery as the first line treatment. In this study 20% patients received adjuvant chemotherapy and/or radiotherapy and prophylactic whole brain radiotherapy (WBRT). They found that in patients who did not receive PCI at median follow up of 23.4 months, 25% developed brain metastasis. At 5 years after diagnosis overall survival was 43% (100). Further trials are needed to clarify the role of PCI in this population. If patients with pulmonary LCNEC are found to have brain metastasis, stereotactic radiosurgery (SRS) is associated with improved survival (102).

There is limited data available on the role of tumor directed radiation and PCI in extra pulmonary LCNEC however in retrospective series fewer brain metastasis are seen in extra pulmonary as compared to pulmonary LCNEC. In one retrospective review of high grade neuroendocrine carcinoma of the colon and rectum (combining both LCNEC and SCNEC) only 2 of 126 patients presented with brain metastasis at diagnosis (94). In another large retrospective study of high grade extra pulmonary neuroendocrine carcinoma (including both LCNEC and SCNEC) only 1.6% of patients had brain metastases at presentation (34). Given the low risk of brain metastases PCI is not routinely done in this patient population.

### Stage IV

In patients who present with advanced pulmonary LCNEC there have been 2 prospective trials examining first line systemic treatment and several retrospective studies **(** **Table 7** **).**

**Table 7:** Key Papers Describing First Line Therapy in Metastatic Pulmonary LCNEC.

Study	Study Design	Patient population	Total Patients	Treatment	Outcomes	Other
Le Treut et al.(103)	Prospective, phase II, single arm study of etoposide and cisplatin	Stage IV/IIIB LCNEC	42 patients (29 patients with LCNEC by centralized pathology review)	Etoposide/Cisplatin	For whole cohort median OS 7.7 months, ORR 38%, 64% DCR, at 1 year OS was 26.8%. LCNEC cases proven on central review, ORR 34%.	
Niho et al. (14)	Prospective, phase II, single arm study of irinotecan and cisplatin	Advanced pulmonary LCNEC	44 patients (30 patients with pure LCNEC)	Irinotecan/ Cisplatin	ORR in pure LCNEC was 46.7%, median OS 12.6 months. 1 year OS was 62.1%, 2 year OS was 18.4%.	
Derks et al. (104)	Retrospective review	Stage IV chemotherapy treated LCNEC patients	207 patients	3 groups of chemotherapy regimens reviewed:	Median OS 7.3 months. NSCLC-t group with median OS of 8.5 months *vs* median OS of 5.9 months in NSCLC-t group and 6.7 months in SCLC-t group.	
				“NSCLC-pt” Platinum based therapy with gemcitabine, docetaxel, paclitaxel or vinorelbline, most platinum/gemcitabine (N=60) “NSCLC-t” Platinum and pemetrexed (N=20) “SCLC-t” Platinum and etoposide (N=64)		
Derks et al. (105)	Retrospective review	Patients with stage IV LCNEC	232 cases (148 confirmed on pathology review, 79 with chemotherapy regimens available)	3 regimens compared:	LCNEC with wild type RB1 gene showed significantly longer OS when treated with platinum with gemcitabine / taxane (9.6 months) as compared to platinum / etoposide (5.8 months).	
				Platinum with gemcitabine / taxane Platinum and etoposide Platinum/ pemetrexed		
Zhuo et al. (106)	Retrospective review	Patients with advanced stage LCNEC, analyzed by molecular subgroups	63 patients (54 patients with advanced stage disease receiving first line chemotherapy)	3 regimens compared:	ORR from all chemotherapy regimens was 46.7% in SCLC- like LCNEC as compared to 25.6% in NSCLC- like LCNEC. In SCLC-like LCNEC RR (75%) to platinum/etoposide was higher than platinum/ pemetrexed (0%). In NSCLC-like LCNEC there was no difference in RR between the three chemotherapy regimens, however platinum/etoposide regimens were associated with longer PFS (5.2 months) than platinum/gemcitabine/taxane (2.5 months).	
				Platinum with gemcitabine / taxane Platinum and etoposide Platinum/ pemetrexed		

RFA, recurrence free survival; OS, overall survival; LCNEC, Large cell neuroendocrine carcinoma; SCLC, Small cell Lung Cancer; DFS, Disease free survival; NSCLC, Non-small cell lung cancer; DCR, Disease control rate; ORR, Overall response rate.

Niho et al., performed a single arm phase II study of irinotecan and cisplatin as first line therapy in advanced pulmonary LCNEC. Of 44 patients enrolled, response rate in the intention to treat group was 54.5%. Median PFS was 5.9 months and median OS was 15.1 months. Upon central pathology review, 20 of these patients had pure LCENC. The response rate in the pure LCNEC group was 46.7%. Median overall survival was 12.6 months in the pure LCNEC group (14). In a second trial Le Treut et al. performed a prospective, single arm phase II study of advanced stage LCNEC treated with cisplatin and etoposide. This study enrolled 42 patients with advanced LCNEC of the lung. Upon central pathologist review, 29 of the 40 patients with tissue available for review were pure LCNEC. The objective response was 38%, with a 64% disease control rate. Median PFS in the intention to treat population was 5.3 months and the median overall survival was 7.7 months (103). Given the small number of patients included in each of these trials and the fact that the two regimens have never been directly compared in randomized control trials, it is hard to draw any specific conclusions about irinotecan and cisplatin *versus* cisplatin and etoposide in this patient population.

Retrospective studies have also sought to explore these questions. One retrospective from the Netherlands examined 207 patients with LCNEC who were treated with 3 different regimens, a platinum (carboplatin, cisplatin) plus either gemcitabine, docetaxel, paclitaxel or vinorelbine (called by the authors NSCLC-t), pemetrexed (called NSCLC-pt), or etoposide (called SCLC-t). They found improved median OS in the NSCLC-t group of 8.4 months, compared to 5.9 months in the NSCLC-pt group and 6.7 months in the SCLC-t group. Specifically, the biggest advantage to the NSCLC-t treatment program was in treatment of a platinum combined with gemcitabine (104). The same group examined 148 patients with LCNEC who had next generation sequencing performed and correlated molecular markers with overall survival and PFS. They also stratified response for NSCLC chemotherapy including platinum and gemcitabine or taxanes *versus* platinum and etoposide combinations. They concluded that patients with LCNEC that carry a wild type *RB1* gene have improved outcomes with platinum and gemcitabine or taxanes (NSCLC regimens) *versus* platinum and episode based treatment (105). This study was limited by its retrospective nature and small patient population. Other groups have sought to stratify LCNEC by novel genomic subtypes described above (43). In a retrospective study of 63 patients with advanced LCNEC patients were stratified by tumor mutation into SCLC-like and NSCLC- like LCNEC based on genomic features from tumor DNA or circulating free DNA. They found treatment with platinum and etoposide was associated with superior response and survival in SCLC-like LCNEC. However, treatment with gemcitabine, taxane and platinum was not associated with improved survival in NSCLC-like LCNEC (106). Additional studies are needed to better evaluate how molecular subtypes of LCNEC influence outcomes.

No prospective clinical trials for second and third line treatments in metastatic pulmonary LCNEC were identified **(** **Table 8** **).** Two retrospective studies examined amrubicin monotherapy as a second line treatment for pulmonary LCNEC. The first study included 18 patients with LCNEC or “high-grade non-small-cell neuroendocrine carcinoma” all of whom had received platinum based first line therapy. The overall response rate to single agent amrubicin was 11.1%. with median PFS of 4.0 and median OS of 9.1 months (114). Another retrospective study also examined amrubicin in advanced pulmonary LCNEC after progression on platinum-based chemotherapy. This study include 18 patients; objective response rate (ORR) was 28%, median PFS was 3.1 months and OS was 5.1 months (115).

**Table 8:** First Line Therapy in Metastatic Extra-Pulmonary LCNEC (most studies report outcomes for cohorts with combined SCNEC and LCNEC).

Study	Study Design	Patient population	Total Patients	Treatment	Outcomes	Other
Li et al. (107)	Prospective Phase II single arm study	Metastatic gastroenteropancreatic neuroendocrine carcinoma	40 (20 SCNEC, 8 LCNEC, 7 MANEC, 5 NET w/ elevated Ki-67)	Irinotecan/Cisplatin followed by octreotide LAR maintenance	32 patients evaluable for tumor response, ORR 45%, DCR 70% Median OS 12.8 months	
Alifieris et al. (108)	Prospective Phase II single arm study	Advanced neuroendocrine carcinoma of the colon or small bowel	22 patients	First line capecitabine, irinotecan, oxaliplatin plus bevacizumab, for 6 cycles, if disease responding or stable, patients received maintenance therapy with pazopanib and capecitabine.	19 patients evaluable, ORR 47.4% (3 CR, 6 PR) Median PFS 13 months, median OS was 29 months	
Mitry et al. (109)	Retrospective review	Gastroenteropancreatic neuroendocrine tumors	52 patients (41 poorly differentiated neuroendocrine carcinoma)	Etoposide/Cisplatin was given in 29/41 of poorly differentiated patients	Median OS for poorly differentiated tumors was 2.3 months ORR for patients with poorly differentiated disease was 41.5%	
Terashima et al. (110)	Retrospective review	Advanced extra-pulmonary neuroendocrine carcinomas	41 patients	18 patients received Etoposide/Cisplatin 22 patients received Irinotecan/Cisplatin	Median OS was 9.2 months Patients who received Etoposide/Cisplatin had median OS of 7.3 months *vs* 14.0 months with Irinotecan/Cisplatin	
Du et al. (111)	Retrospective review	High-grade gastrointestinal neuroendocrine neoplasm	11 (included both primary metastatic disease and patients who recurred after initial therapy)	Irinotecan combined with 5-fluorouracil and leucovorin	PR in 7 patients (ORR 63.6%) Median OS was 13.0 months	
Yamaguchi et al. (112)	Retrospective review	Unresectable or recurrent neuroendocrine carcinomas of the digestive system	258, 206 patients received first line irinotecan (N=160) or etoposide (N=46) based regimens	Variable, most common regimens irinotecan/cisplatin, etoposide/cisplatin, and 5-FU based regimens	Median OS was 11.5 months ORR in irinotecan/cisplatin 50%, median OS 13 months, etoposide/cisplatin ORR 28%, median OS 7.3 months	
Bongiovanni etl a. (113)	Retrospective review	Metastatic gastroenteropancreatic neuroendocrine carcinoma	19 patients (13 SCNEC, 7 LCNEC)	Most common regimen etoposide/cisplatin	Median OS was 13.5 months Patients with BMI ≥25 kg/m2 had a poor prognosis as did patients with Ki-67 >55%	

NET, Neuroendocrine tumor; OS, overall survival; LCNEC, Large cell neuroendocrine carcinoma; SCNEC, Small cell neuroendocrine carcinoma; Long-acting release, LAR; MANEC, mixed adenoneuroendocrine carcinoma; BMI, Body mass index; ORR, overall response rate.

In advanced extra-pulmonary LCNEC two prospective studies have evaluated treatment in GI HGNEC **(** **Table 9** **).** Li et al. completed a prospective, single arm phase II study of irinotecan and cisplatin followed by octreotide LAR maintenance. This study included 8 LCNEC patients. In total ORR was 45% and median OS was 12.8 months (107). A recent two-stage phase II study was done to evaluate the efficacy and safety of a first line capecitabine, oxaliplatin, irinotecan, and bevacizumab (CAPOXIRI-BEV) combination followed by pazopanib plus capecitabine maintenance therapy in patients with advanced GI HGNEC. In the 22 patients enrolled, ORR was 47.7% and overall disease control rate was 78.9%. Median PFS was 13 months and median OS was 29 months. Subcategories of LCNEC *versus* SCLC were not reported (108). Currently a randomized phase II trial (the SENECA trial) of folinic acid, 5-fluorouracil and irinotecan (FOLFIRI) or capecitabine plus temozolomide (CAPTEM) as 2^nd^ line therapy in advanced extra-pulmonary HGNEC is being planned to better understand this topic (117).

**Table 9:** Key Papers Describing 2^nd^/3^rd^ Line treatments in Metastatic Pulmonary LCNEC.

Study	Study Design	Patient population	Total Patients	Treatment	Outcomes	Other
Yoshida et al. (115)	Retrospective review	Advanced LCNEC who received 1 (N=13) or 2 (N=5) prior chemotherapy regimens, most received platinum based therapy	18 patients	2^nd^ or 3^rd^ line amrubicin monotherapy	17 patients were evaluable, ORR 27.7%, median OS 5.1 months	
Shimada et al. (116)	Retrospective review	LCNEC or high-grade neuroendocrine carcinoma probably LCNEC, who underwent chemotherapy as initial therapy	25 patients (12 patients received 2^nd^ line chemotherapy	Variable	ORR to 2^nd^ line therapy was 17% 1 year OS rate was 34%	
Kasahara et al. (114)	Retrospective review	Advanced high−grade non−small−cell neuroendocrine carcinoma or LCNEC, received first line platinum based therapy	18 patients	2^nd^ line amrubicin monotherapy	ORR 11.1%, DCR 61.1% Median OS was 9.1 months Irinotecan monotherapy was the most frequently used 3^rd^ line treatment	

OS, overall survival; LCNEC, Large cell neuroendocrine carcinoma; SCNEC, Small cell neuroendocrine carcinoma; ORR, overall response rate; DCR, Disease control rate.

There are several retrospective studies examining efficacy of first line chemotherapy in metastatic GI HGNEC. The efficacy of platinum-based chemotherapy was retrospectively reviewed in 20 patients with GI HGNEC. Of the 20 patients in this review, 7 had LCNEC, and all received both FDG PET/CT scan and Gallium 68 DOTATATE PET/CT scans. Of included patients, 80% had positive FDG PET/CT scans and 35% had positive Gallium 68 DOTATATE PET/CT scans. In this study 75% of patients received cisplatin and etoposide as first line treatment and 25% received carboplatin and etoposide. The ORR was 68% and median OS was 13.5 months. Interestingly, 2 patients received peptide receptor radionuclide therapy (PRRT) as second line treatment (113). A review of the national cancer database of patients with HGNECs of the colon and rectum was performed. 1208 cases were identified, with 46.7% of patients presenting with metastatic disease. By multivariable analysis, resection, chemotherapy, and rectal primary site were associated with better OS. Median OS in this study was 9.0 months (40).

There is limited retrospective literature for second line treatment in advanced GI HGNEC **(** **Table 10** **)**. A systematic review and meta-analysis by McNamara et al. reviewed second line chemotherapy in advanced extra pulmonary poorly differentiated neuroendocrine carcinoma including both SCLC and LCNEC. This review identified 19 studies with 4 prospective studies (2 of which do not yet have available results) and 15 retrospective studies with 582 patients. When all studies were taken together median response rate was 18%. The ORR was 0% for single agent first line therapies everolimus, temozolomide, and topotecan. The ORR was 50% for amrubicin. In the whole cohort median PFS was 2.5 months, median OS was 7.64 months (121). Amrubicin was also retrospectively studied in another study as a monotherapy for patients with platinum refractory disease. In this study, 10 patients with progression on platinum based therapy for metastatic GI HGNEC were included who received second line amrubicin monotherapy. In this very small study 2 patients had a partial response for an ORR of 20% (118).

**Table 10:** Key Papers Describing 2^nd^/3^rd^ Line treatments in Metastatic Extra-Pulmonary LCNEC (most studies report outcomes for cohorts with combined SCNEC and LCNEC).

Study	Study Design	Patient population	Total Patients	Treatment	Outcomes	Other
Ando et al. (118)	Retrospective review	Advanced gastroenteropancreatic neuroendocrine carcinoma, first line therapy with platinum based chemotherapy	10 patients	2^nd^ line amrubicin monotherapy	ORR 20% Median OS 5.0 months	
Hadoux et al. (119)	Retrospective review	Advanced poorly differentiated grade 3 neuroendocrine carcinoma, first line therapy with platinum based chemotherapy (combined all sites including 2 bronchus tumors, majority were GI primary sites)	20 patients (12 LCNEC, 7 SCNEC, 1 unknown)	2^nd^ (N= 12) or 3^rd^ line or greater (N=8) treatment with FOLFOX	17 patients were evaluable, ORR 29% Median OS 9.9 months	
Nio et al. (120)	Retrospective review	GI extra-pulmonary neuroendocrine carcinoma, first line therapy with platinum based chemotherapy	13 patients	2^nd^ line amrubicin monotherapy	ORR 38.5% Median OS 7.1 months	

GI, Gastrointestinal; OS, overall survival; LCNEC, Large cell neuroendocrine carcinoma; SCNEC, Small cell neuroendocrine carcinoma; ORR, overall response rate.

One study retrospectively examined 5-fluorouracil and oxaliplatin (FOLFOX) as second line treatment for high grade neuroendocrine carcinoma. The study included 20 patients, 12 LCNEC, 7 SCNEC and 1 unknown primary; 12 of the 20 patients had tumors of gastroenteropancreatic origin. In this study, 12 patients received FOLFOX as second line treatment and 8 as third line. Median PFS was 3.5 months, median OS was 9.9 months, ORR was 29% (119). In GI HGNEC after progression on platinum with etoposide or irinotecan, promising other agents include 5-fluorouracil based regimens and amrubicin. There will likely also be a role for immunotherapy in this population moving forward, however additional studies are needed.

## Future Directions

Although atezolizumab plus carboplatin and etoposide has been established as the first line therapy in SCLC (122), the role of immunotherapy has not been clearly established in LCNEC. Most of the available data for the use of immunotherapy agents in this population are from small retrospective case series and case reports. Levra et al. reported an impressive ORR of 60% in a case series of 10 patients with advanced pulmonary LCNEC treated with single agent nivolumab or pembrolizumb as a second line therapy. In this study median PFS was 57 weeks (123). Other case reports have shown response to nivolumab in patients with pulmonary LCNEC (124, 125). A recent larger retrospective study reviewed 37 consecutive patients with advanced pulmonary LCNEC. The patients were divided into two groups N=23 patients treated with immunotherapy and N=14 patients not treated with immunotherapy. In the group treated with immunotherapy ORR was 33% and median PFS 4.2 months, median OS with immunotherapy was 11.8 months. These outcomes were very similar to response to immunotherapy in NSCLC (126). The Dual Anti-CTLA-4 and Anti-PD-1 Blockade in Rare Tumors (DART) trial is a prospective, open-label, multicenter phase II clinical trial of ipilimumab plus nivolumab across multiple rare tumor cohorts. Patients were eligible if they had advanced non-pancreatic neuroendocrine tumors with progression following at least one line of standard systemic therapy. The neuroendocrine cohort included 18 patients with HGNEC with gastrointestinal or lung primaries. The breakdown of histologic subgroups, including the specific number of patients with LCNEC was not reported. For the group of all HGNEC patients ORR was 44% (127). Although these studies series do suggest potential responses to immunotherapy in LCNEC, larger studies are needed to both determine benefit of immunotherapy as a monotherapy or in combination with chemotherapy and to establish which patients benefit most from immunotherapy treatment.

Another important area for study is biomarkers for response to immunotherapy in LCNEC. As reviewed above, PD-L1 is an important marker of response to immunotherapy in other cancer types, the role in LCNEC is not yet defined. A recent retrospective study reviewed 13 patients with pulmonary LCNEC treated with either pembrolizuamb or nivolumab. In this study the ORR for anti-PD-1 therapy was 39% and OS was 13.7 months. Perhaps most interestingly among the 13 patients treated with immunotherapy 10 patients had PD-L1 negative tumors. The ORR among patients with negative PD-L1 expression was 40%. High density of CD8 positive tumor infiltrating lymphocytes (TILs) (≥38/mm2) was associated with improved ORR. Response to immunotherapy was associated with *TP53* mutation co-occurring with either *PIK3CA* or *RB1* (128). Further larger clinical trials are needed to evaluate whether PD-L1 status will be a useful marker for LCNEC response to biotherapy or if molecular characteristics of these tumors can help guide treatment to anti-PD-1 therapies. Circulating tumor cells (CTCs) and cell free DNA (cfDNA) are used as liquid biopsy biomarkers in solid tumors to monitor and assess response in recurrent or metastatic disease and may have a role in identifying patients who could respond to immunotherapy in LCNEC (129). One recent study evaluated blood T cell repertoire alterations in stage IV pulmonary LCNEC patients enrolled in a trial of carboplatin and paclitaxel. Patients with pulmonary LCNEC had significant T-cell repertoire alterations (p < 0.001) compared to age-matched smokers. Increased T cell repertoire alterations were associated with better treatment response and longer survival (130). Assessment of T cells in the peripheral blood may also play a future role in prognostication and treatment selection of LCNEC patients.

As stated above median TMB in pulmonary LCNEC has been reported to be 9.9 mutations/Mb (56) and 5.68 mutations/Mb (67) in GI HGNEC. Use of TMB as marker of response to combined checkpoint inhibition was evaluated in SCLC in the CheckMate 032 trial. This randomized, multicenter, open-label, phase 1/2 trial of nivolumab ± ipilimumab evaluated nivolumab monotherapy and nivolumab plus ipilimumab in advanced SCLC after progression on prior therapy. Although not statistically significant the results suggested a potential survival benefit from nivolumab plus ipilimumab *versus* nivolumab monotherapy for patients with a high TMB (131). Further studies are needed to better determine prevalence and clinical utility of elevated TMB in LCNEC.

The KEYNOTE-158 study is perhaps most notable for evaluating response to pembrolizumab in patients with elevated tumor mutation burden (TMB) which led to the FDA approval of pembrolizumab for this indication. In this study elevated TMB was defined as least 10 mutations per megabase. Patients with elevated TMB above this cutoff had improved response to pembrolizumab monotherapy (132). It is also important to note that a definition of TMB high as 10 mutations per megabase may not be the most meaningful measure in all tumor types. The TMB cutoffs that is associated with improved overall survival with immunotherapy varies between cancer subtypes. One study evaluated cut off of TMB based on the top 20% of normalized mutation burden for each cancer type. For example in renal cell carcinoma cutoff was found to be 5.9 while in colorectal cancer the cutoff was 52.2 (133). The cutoff for elevated TMB with benefit with immunotherapy in LCNEC is not known.

A retrospective cohort study of 2589 patients examined rare solid tumors including neuroendocrine tumors for elevated TMB (using the same cutoff of KEYNOTE-158 of 10 mutations per Mb). They reviewed the same rare tumor types as in KEYNOTE-158 and found that prevalence of high TMB was highest in SCLC in 122 of 305 patients (40%) and in NETs in 48 of 164 tumors (29.3%) (134). They do not provide breakdown of the NET patients by WHO grading criteria, however a note does mention that of 164 pts with NETs 90 had “lung large cell NET” and half of these patients had elevated TMB. One might speculate that a portion of these patients in fact had LCNEC rather than carcinoid or atypical carcinoid tumors.

Recently several promising future therapy directions have been reviewed in SCLC including Poly [ADP-ribose] polymerase (PARP) inhibitors in combination with chemotherapy and targeting of histone-lysine N-methyltransferase EZH2 (135). PARP inhibitors may also have a role in LCNEC as LCNEC has been found to have elevated PARP1 in review of IHC of LCNECs. However, strong PARP1 intensity was more frequent in SCLC, in 87.5% of samples *versus* in 67.6% of LCNEC samples (136). Similarly EZH2 expression is also elevated in LCNEC and agents targeting EZH2 may be useful in LCNEC (137).

Another promising target for future immunotherapy research is delta like protein 3 (DLL3) a Notch family receptor involved in the achaete scute complex-like 1 (ASCL1) pathway. In murine models, overexpression of DLL3 was associated with cell proliferation, tumor growth and reduced apoptosis (138). A retrospective study of 94 patients with metastatic pulmonary LCNEC evaluated DLL3 expression. DLL3 expression was considered positive if ≥ 1% of tumor cells showed cytoplasmic/membranous or dot-like immunostaining. They found positive DLL3 in 70/93 (74%) samples (139). DLL3 is also expressed in GI HGNEC. One study reviewed DLL3 expression in GI HGNEC and found strong expression of DLL3 in patient tissue samples with levels comparable to SCLC (140).

DLL3 expression is a potential therapeutic target for DLL3 targeting agent. One such agent is Rovalpituzumab-tesirine (Rova-T) a DLL3 targeting antibody drug conjugate (139, 141, 142). Rova-T was evaluated in a phase 1 study and then a phase 2 study which confirmed antitumor activity but was notable for a high percentage of a patients with adverse events (141, 143). A new formulation was recently developed called SC-002 which uses a different linker leading to a more uniform drug antibody ratio, which was thought to improve safety. A phase I study of this new formation was done and included 35 patients all with DLL3-high (≥75%) expressing SCLC or LCNEC. In this study 66% of patients experienced serious adverse events, of which 37% were considered related to the drug. However, 14% of patients achieved partial response (144). Further research is needed to evaluate whether DLL3 -targeted antibody-drug conjugates can be used in this patient population. In addition to antibody-drug conjugates, bispecific T cell engagers and chimeric antigen receptor T cell therapy targeting DLL3 are also in development (145).

Aurora kinase inhibitors are another potential target in SCLC which are also promising in LCNEC. A recent study examined subgroups of LCNEC including subtypes defined by TTF-1 and c-Myc, which have been previously described as clinically relevant subgroups in SCLC. They also examined DLL3 in this patient population. IHC of lobectomy and biopsy specimens was reviewed for 27 patients with pulmonary LCNEC. Positivity for c-MYC was defined as cases where ≥40% tumors cells reacted with any intensity; positivity for TTF-1 expression was defined cases where >10% of the tumor cells reacted with any intensity. The authors identified 10 cases with only TTF-1 and 15 cases with only c-MYC present, 2 cases were positive for both. DLL3 was found in 15 patients and was associated with TTF-1 expression. They conclude that LCNEC, similar to SCLC can be further divided into subgroups by TTF-1 and c-MYC expression (146). This has important treatment implications as c-MYC expression is associated with aurora kinase expression and may indicate responsiveness to aurora kinase inhibitors (147). Further studies should include biomarker correlates to help identify subgroups of LCNEC that may best respond to specific treatments.

Somatostatin targeting agents including somatostatin analogues and Peptide Receptor Radionuclide Therapy (PRRT) are effective in well-differentiated neuroendocrine tumors. Research is ongoing as to determine the efficacy in HGNEC including LCNEC. Case reports describe promising response to PRRT in patients with pulmonary LCNEC with positive somatostatin receptor scintigraphy (148). Immunohistochemical analysis of SSTR-2 expression in pulmonary neuroendocrine tumors was reviewed retrospectively in a series of 218 “clinically aggressive cases” including 60 LCNEC. They found that SSTR-2 was expressed in 33% of LCNEC and 38% of SCLC (73). Another study reviewed immunohistochemical analysis of SSTR-2 expression of GI HGNEC including 142 patients with LCNEC. In the LCNEC group, 22.1% of these patients had strongly positive expression of SSTR-2 (72). An analysis of somatostatin receptor scintigraphy in pulmonary LCNEC was done in 26 patients with technetium-99m ethylene diamine-diacetic acid/hydrazinonicotinyl-Tyr3-octreotide (Tc-TOC). This study found that 100% of primary lesions showed uptake of Tc-TOC while 84% of metastases were visualized (as compared to conventional imaging) (149). Another study retrospectively examined 26 patients with primary pulmonary LCNEC and compared them to 40 patients with adenocarcinoma. In this study, 50% of patients had high expression of somatostatin receptors (150). Another retrospective study examined 18 consecutive patients with resected pulmonary LCNEC who underwent preoperative indium In-111 pentetreotide scintigraphy. Of this group, 10 patients (55.5%) had positive OctreoScan scans. In this study all patients with TNM pathologic stage higher than Ib received post-operative radiotherapy. Those with positive SRS avidity received post-operative octreotide LAR; of these patients 9 were still alive and disease free at the time the study was published (151). Further studies are needed to better understand the role of SSTR-2 directed therapy in LCNEC.

## Conclusion

LCNEC are rare and aggressive tumors, and current evidence suggests surgery for local disease with platinum and etoposide or platinum and irinotecan based adjuvant chemotherapy is the most effective treatment. In advanced disease, the historical regimen of platinum based therapy in combination with etoposide or irinotecan remains among the commonly used first line therapies, however for extra thoracic LCNEC regimens like FOLFOX, FOLFOIRI and CAPTEM can also be used. Further effective and safe treatment options are desperately needed.

## Author Contributions

These authors have contributed equally to this work, VC, SA, LA. This author is senior author, AC. All authors contributed to the article and approved the submitted version.

## Conflict of Interest

AC has received research support in form of either grant or drug supply from BMS, Clovis, Lexicon, Nanopharmaceuticals, EMD Serono, ECS Progastrin. AC is also an advisor to Ipsen, Lexicon, Novartis, TerSera but has not accepted direct personal payments for advisory role. VC owns equity in Pfizer, BristolMyers Squibb, Seagen, and Viatris.

The remaining authors declare that the research was conducted in the absence of any commercial or financial relationships that could be construed as a potential conflict of interest.

## Publisher’s Note

All claims expressed in this article are solely those of the authors and do not necessarily represent those of their affiliated organizations, or those of the publisher, the editors and the reviewers. Any product that may be evaluated in this article, or claim that may be made by its manufacturer, is not guaranteed or endorsed by the publisher.

## References

[1] Strosberg JR Coppola D Klimstra DS Phan AT Kulke MH Wiseman GA. The NANETS Consensus Guidelines for the Diagnosis and Management of Poorly Differentiated (High-Grade) Extrapulmonary Neuroendocrine Carcinomas. Pancreas (2010) 39(6):799–800. 10.1097/MPA.0b013e3181ebb56f 20664477 PMC3100733

[2] Burkeen G Chauhan A Agrawal R Raiker R Kolesar J Anthony L. Gynecologic Large Cell Neuroendocrine Carcinoma: A Review. Rare Tumors (2020) 12:2036361320968401. 10.1177/2036361320968401 33194158 PMC7605029

[3] Tu X Chang T Nie L Qiu S Xu H Huang Y. Large Cell Neuroendocrine Carcinoma of the Prostate: A Systematic Review and Pooled Analysis. Urol Int (2019) 103(4):383–90. 10.1159/000499883 30965328

[4] Thompson ED Stelow EB Mills SE Westra WH Bishop JA. Large Cell Neuroendocrine Carcinoma of the Head and Neck: A Clinicopathologic Series of 10 Cases With an Emphasis on HPV Status. Am J Surg Pathol (2016) 40(4):471–8. 10.1097/PAS.0000000000000580 PMC4792746 26735857

[5] Ogata S Maeda R Tomita M Sato Y Ayabe T Nakamura K. Resected Thymic Large Cell Neuroendocrine Carcinoma: A Case Report and Review of the Literature. Int J Surg Case Rep (2019) 60:53–7. 10.1016/j.ijscr.2019.05.057 PMC6580310 31202999

[6] Xia K Zhong W Chen J Lai Y Huang G Liu H. Clinical Characteristics, Treatment Strategy, and Outcomes of Primary Large Cell Neuroendocrine Carcinoma of the Bladder: A Case Report and Systematic Review of the Literature. Front Oncol (2020) 10:1291. 10.3389/fonc.2020.01291 32850401 PMC7399333

[7] Fernandez FG Battafarano RJ. Large-Cell Neuroendocrine Carcinoma of the Lung: An Aggressive Neuroendocrine Lung Cancer. Semin Thorac Cardiovasc Surg (2006) 18(3):206–10. 10.1053/j.semtcvs.2006.08.007 17185180

[8] Kinslow CJ May MS Saqi A Shu CA Chaudhary KR Wang TJC. Large-Cell Neuroendocrine Carcinoma of the Lung: A Population-Based Study. Clin Lung Cancer (2020) 21(2):e99–113. 10.1016/j.cllc.2019.07.011 31601526

[9] Deng C Wu S-G Tian Y. Lung Large Cell Neuroendocrine Carcinoma: An Analysis of Patients From the Surveillance, Epidemiology, and End-Results (SEER) Database. Med Sci Monit (2019) 25:3636–46. 10.12659/MSM.914541 PMC6537662 31095532

[10] Korse CM Taal BG van Velthuysen M-LF Visser O. Incidence and Survival of Neuroendocrine Tumours in the Netherlands According to Histological Grade: Experience of Two Decades of Cancer Registry. Eur J Cancer (2013) 49(8):1975–83. 10.1016/j.ejca.2012.12.022 23352435

[11] Cao L Li Z-W Wang M Zhang T-T Bao B Liu Y-P. Clinicopathological Characteristics, Treatment and Survival of Pulmonary Large Cell Neuroendocrine Carcinoma: A SEER Population-Based Study. PeerJ (2019) 7:e6539. 10.7717/peerj.6539 30944773 PMC6441320

[12] Travis WD Linnoila RI Tsokos MG Hitchcock CL Cutler GB Nieman L. Neuroendocrine Tumors of the Lung With Proposed Criteria for Large-Cell Neuroendocrine Carcinoma. An Ultrastructural, Immunohistochemical, and Flow Cytometric Study of 35 Cases. Am J Surg Pathol (1991) 15(6):529–53. 10.1097/00000478-199106000-00003 1709558

[13] Travis WD Brambilla E Nicholson AG Yatabe Y Austin JHM Beasley MB. The 2015 World Health Organization Classification of Lung Tumors: Impact of Genetic, Clinical and Radiologic Advances Since the 2004 Classification. J Thorac Oncol (2015) 10(9):1243–60. 10.1097/JTO.0000000000000630 26291008

[14] Niho S Kenmotsu H Sekine I Ishii G Ishikawa Y Noguchi M. Combination Chemotherapy With Irinotecan and Cisplatin for Large-Cell Neuroendocrine Carcinoma of the Lung: A Multicenter Phase II Study. J Thorac Oncol (2013) 8(7):980–4. 10.1097/JTO.0b013e31828f6989 23774385

[15] WHO Classification of Tumours Editorial Board. WHO Classification of Tumours of the Lung, Pleura, Thymus and Heart (Medicine). 4th ed., eds. Travis WD Brambilla E Burke AP Marx A Nicholson AG Lyon: World Health Organization (2015). 10.1097/JTO.0000000000000663 26291007

[16] Zhang J-T Li Y Yan L-X Zhu Z-F Dong X-R Chu Q. Disparity in Clinical Outcomes Between Pure and Combined Pulmonary Large-Cell Neuroendocrine Carcinoma: A Multi-Center Retrospective Study. Lung Cancer (2020) 139:118–23. 10.1016/j.lungcan.2019.11.004 31775086

[17] Verma R Lambert A Katz HH Benson SJ. Ectopic ACTH-Producing Large Cell Neuroendocrine Pancoast Tumour Presenting as Horner Syndrome. BMJ Case Rep (2017) 2017. 10.1136/bcr-2016-219156 PMC5372167 28343156

[18] Saito T Kimoto M Nakai S Ikoma A Toyoshima H Kawakami M. Ectopic ACTH Syndrome Associated With Large Cell Neuroendocrine Carcinoma of the Thymus. Intern Med (2011) 50(14):1471–5. 10.2169/internalmedicine.50.5160 21757832

[19] Lin D Suwantarat N Kwee S Miyashiro M. Cushing’s Syndrome Caused by an ACTH-Producing Large Cell Neuroendocrine Carcinoma of the Gallbladder. World J Gastrointest Oncol (2010) 2(1):56–8. 10.4251/wjgo.v2.i1.56 PMC2999155 21160818

[20] Oda N Miyahara N Tabata M Minami D Ninomiya K Kanehiro A. Pneumocystis Pneumonia Concomitant With Ectopic ACTH Syndrome Caused by a Large Cell Neuroendocrine Carcinoma of the Thymus. Intern Med (2017) 56(5):551–5. 10.2169/internalmedicine.56.7655 PMC5399209 28250304

[21] Nakamura T Fujisaka Y Tamura Y Tsuji H Matsunaga N Yoshida S. Large Cell Neuroendocrine Carcinoma of the Lung With Cancer-Associated Retinopathy. Case Rep Oncol (2015) 8(1):153–8. 10.1159/000380943 PMC4386109 25873883

[22] Stanford MR Edelsten CE Hughes JD Sanders MD Brooks CI Mitchell D. Paraneoplastic Retinopathy in Association With Large Cell Neuroendocrine Bronchial Carcinoma. Br J Ophthalmol (1995) 79(6):617–8. 10.1136/bjo.79.6.617-a PMC505178 7626582

[23] Mitchell AN Bakhos CT Zimmerman EA. Anti-Ri-Associated Paraneoplastic Brainstem Cerebellar Syndrome With Coexisting Limbic Encephalitis in a Patient With Mixed Large Cell Neuroendocrine Lung Carcinoma. J Clin Neurosci (2015) 22(2):421–3. 10.1016/j.jocn.2014.06.103 25443085

[24] Grommes C Preston DC Al-Kadhimi Z Alshekhlee A. Lambert-Eaton Syndrome With Large-Cell Neuroendocrine Carcinoma of the Lung. Muscle Nerve (2008) 37(6):786–9. 10.1002/mus.21032 18506722

[25] Demirer T Ravits J Aboulafia D. Myasthenic (Eaton-Lambert) Syndrome Associated With Pulmonary Large-Cell Neuroendocrine Carcinoma. South Med J (1994) 87(11):1186–9. 10.1097/00007611-199411000-00030 7973914

[26] Oh HJ Lee MJ Jang SJ Shin DH Kang S-W. Syndrome of Inappropriate Antidiuretic Hormone Secretion in a Patient With Large Cell Neuroendocrine Carcinoma. Yonsei Med J (2012) 53(3):667–9. 10.3349/ymj.2012.53.3.667 PMC3343429 22477016

[27] Rindi G Petrone G Inzani F. The 2010 WHO Classification of Digestive Neuroendocrine Neoplasms: A Critical Appraisal Four Years After Its Introduction. Endocr Pathol (2014) 25(2):186–92. 10.1007/s12022-014-9313-z 24699927

[28] Nagtegaal ID Odze RD Klimstra D Paradis V Rugge M Schirmacher P. The 2019 WHO Classification of Tumours of the Digestive System. Histopathology (2020) 76(2):182–8. 10.1111/his.13975 PMC7003895 31433515

[29] Kim JY Hong S-M Ro JY. Recent Updates on Grading and Classification of Neuroendocrine Tumors. Ann Diagn Pathol (2017) 29:11–6. 10.1016/j.anndiagpath.2017.04.005 28807335

[30] Limonnik V Abel S Finley GG Long GS Wegner RE. Factors Associated With Treatment Receipt and Overall Survival for Patients With Locally Advanced Large Cell Neuroendocrine Carcinoma of the Lung: A National Cancer Database Analysis. Lung Cancer (2020) 150:107–13. 10.1016/j.lungcan.2020.10.001 33126090

[31] Derks JL Hendriks LE Buikhuisen WA Groen HJM Thunnissen E van Suylen R-J. Clinical Features of Large Cell Neuroendocrine Carcinoma: A Population-Based Overview. Eur Respir J (2016) 47(2):615–24. 10.1183/13993003.00618-2015 26541538

[32] Park MS Kim KD Chung JH Shin DH Chung KY Kim JH. Clinical Features of Pulmonary Large Cell Neuroendocrine Carcinoma. Cancer Res Treat (2003) 35(3):245–53. 10.4143/crt.2003.35.3.245 26680943

[33] Zou L Guo T Ye L Zhou Y Chu L Chu X. Outcomes for Surgery in Stage IA Large Cell Lung Neuroendocrine Compared With Other Types of Non-Small Cell Lung Cancer: A Propensity Score Matching Study Based on the Surveillance, Epidemiology, and End Results (SEER) Database. Front Oncol (2020) 10:572462. 10.3389/fonc.2020.572462 33324549 PMC7727448

[34] Alese OB Jiang R Shaib W Wu C Akce M Behera M. High-Grade Gastrointestinal Neuroendocrine Carcinoma Management and Outcomes: A National Cancer Database Study. Oncologist (2019) 24(7):911–20. 10.1634/theoncologist.2018-0382 PMC6656466 30482824

[35] Schmitz R Mao R Moris D Strickler JH Blazer DG. Impact of Postoperative Chemotherapy on the Survival of Patients With High-Grade Gastroenteropancreatic Neuroendocrine Carcinoma. Ann Surg Oncol (2021) 28(1):114–20. 10.1245/s10434-020-08730-0 32556871

[36] Hage R Seldenrijk K de Bruin P van Swieten H van den Bosch J. Pulmonary Large-Cell Neuroendocrine Carcinoma (LCNEC). Eur J Cardiothorac Surg (2003) 23(4):457–60. 10.1016/s1010-7940(02)00837-0 12694759

[37] Grand B Cazes A Mordant P Foucault C Dujon A Guillevin EF. High Grade Neuroendocrine Lung Tumors: Pathological Characteristics, Surgical Management and Prognostic Implications. Lung Cancer (2013) 81(3):404–9. 10.1016/j.lungcan.2013.05.008 23769675

[38] Conte B George B Overman M Estrella J Jiang Z-Q Mehrvarz Sarshekeh A. High-Grade Neuroendocrine Colorectal Carcinomas: A Retrospective Study of 100 Patients. Clin Colorectal Cancer (2016) 15(2):e1–7. 10.1016/j.clcc.2015.12.007 PMC4885752 26810202

[39] Dasari A Mehta K Byers LA Sorbye H Yao JC. Comparative Study of Lung and Extrapulmonary Poorly Differentiated Neuroendocrine Carcinomas: A SEER Database Analysis of 162,983 Cases. Cancer (2018) 124(4):807–15. 10.1002/cncr.31124 PMC5801102 29211313

[40] Fields AC Lu P Vierra BM Hu F Irani J Bleday R. Survival in Patients With High-Grade Colorectal Neuroendocrine Carcinomas: The Role of Surgery and Chemotherapy. Ann Surg Oncol (2019) 26(4):1127–33. 10.1245/s10434-019-07203-3 PMC6402804 30706232

[41] Hermans BCM de Vos-Geelen J Derks JL Latten L Liem IH van der Zwan JM. Unique Metastatic Patterns in Neuroendocrine Neoplasms of Different Primary Origin. Neuroendocrinology (2020). 10.1159/000513249 33227805

[42] Derks JL Leblay N Lantuejoul S Dingemans A-MC Speel E-JM Fernandez-Cuesta L. New Insights Into the Molecular Characteristics of Pulmonary Carcinoids and Large Cell Neuroendocrine Carcinomas, and the Impact on Their Clinical Management. J Thorac Oncol (2018) 13(6):752–66. 10.1016/j.jtho.2018.02.002 29454048

[43] George J Walter V Peifer M Alexandrov LB Seidel D Leenders F. Integrative Genomic Profiling of Large-Cell Neuroendocrine Carcinomas Reveals Distinct Subtypes of High-Grade Neuroendocrine Lung Tumors. Nat Commun (2018) 9(1):1048. 10.1038/s41467-018-03099-x 29535388 PMC5849599

[44] Lu S Stein JE Rimm DL Wang DW Bell JM Johnson DB. Comparison of Biomarker Modalities for Predicting Response to PD-1/PD-L1 Checkpoint Blockade: A Systematic Review and Meta-Analysis. JAMA Oncol (2019). 10.1001/jamaoncol.2019.1549 PMC6646995 31318407

[45] Hermans BCM Derks JL Thunnissen E van Suylen RJ den Bakker MA Groen HJM. Prevalence and Prognostic Value of PD-L1 Expression in Molecular Subtypes of Metastatic Large Cell Neuroendocrine Carcinoma (LCNEC). Lung Cancer (2019) 130:179–86. 10.1016/j.lungcan.2019.02.022 30885341

[46] Abdel Karim N Sendilnathan A Eldessouki I Orr-Asman M Xie C Wang J. PS06.06 Immune Checkpoint Markers in Lung Large Cell Neuroendocrine Carcinomas (L- LCNEC). J Thorac Oncol (2017) 12(11):S1583–4. 10.1016/j.jtho.2017.09.087

[47] Arpin D Charpentier M-C Bernardi M Monnet I Boni A Watkin E. PD-L1-Expression Patterns in Large-Cell Neuroendocrine Carcinoma of the Lung: Potential Implications for Use of Immunotherapy in These Patients: The GFPC 03-2017 “EPNEC” Study. Ther Adv Med Oncol (2020) 12:1758835920937972. 10.1177/1758835920937972 32684990 PMC7343361

[48] Eichhorn F Harms A Warth A Muley T Winter H Eichhorn ME. PD-L1 Expression in Large Cell Neuroendocrine Carcinoma of the Lung. Lung Cancer (2018) 118:76–82. 10.1016/j.lungcan.2018.02.003 29572007

[49] Guleria P Kumar S Malik PS Jain D. PD-L1 Expression in Small Cell and Large Cell Neuroendocrine Carcinomas of Lung: An Immunohistochemical Study With Review of Literature. Pathol Oncol Res (2020) 26(4):2363–70. 10.1007/s12253-020-00832-0 32506394

[50] Inamura K Yokouchi Y Kobayashi M Ninomiya H Sakakibara R Nishio M. Relationship of Tumor PD-L1 (CD274) Expression With Lower Mortality in Lung High-Grade Neuroendocrine Tumor. Cancer Med (2017) 6(10):2347–56. 10.1002/cam4.1172 PMC5633594 28925087

[51] Kasajima A Ishikawa Y Iwata A Steiger K Oka N Ishida H. Inflammation and PD-L1 Expression in Pulmonary Neuroendocrine Tumors. Endocr Relat Cancer (2018) 25(3):339–50. 10.1530/ERC-17-0427 29326364

[52] Kim HS Lee JH Nam SJ Ock C-Y Moon J-W Yoo CW. Association of PD-L1 Expression With Tumor-Infiltrating Immune Cells and Mutation Burden in High-Grade Neuroendocrine Carcinoma of the Lung. J Thorac Oncol (2018) 13(5):636–48. 10.1016/j.jtho.2018.01.008 29378266

[53] Ohtaki Y Kaira K Atsumi J Nagashima T Kawashima O Ibe T. Prognostic Significance of PD-L1 Expression and Tumor Infiltrating Lymphocytes in Large Cell Neuroendocrine Carcinoma of Lung. Am J Transl Res (2018) 10(10):3243–53. PMC6220228 30416665

[54] Tsuruoka K Horinouchi H Goto Y Kanda S Fujiwara Y Nokihara H. PD-L1 Expression in Neuroendocrine Tumors of the Lung. Lung Cancer (2017) 108:115–20. 10.1016/j.lungcan.2017.03.006 28625622

[55] Rekhtman N Pietanza MC Hellmann MD Naidoo J Arora A Won H. Next-Generation Sequencing of Pulmonary Large Cell Neuroendocrine Carcinoma Reveals Small Cell Carcinoma-Like and Non-Small Cell Carcinoma-Like Subsets. Clin Cancer Res (2016) 22(14):3618–29. 10.1158/1078-0432.CCR-15-2946 PMC4995776 26960398

[56] Chae YK Tamragouri K Chung J Schrock AB Kolla B Ganesan S. Genomic Alterations (GA) and Tumor Mutational Burden (TMB) in Large Cell Neuroendocrine Carcinoma of Lung (L-LCNEC) as Compared to Small Cell Lung Carcinoma (SCLC) as Assessed *via* Comprehensive Genomic Profiling (CGP). J Clin Oncol (2017) 35(15_suppl):8517–7. 10.1200/JCO.2017.35.15_suppl.8517

[57] Alcala N Leblay N Gabriel AAG Mangiante L Hervas D Giffon T. Integrative and Comparative Genomic Analyses Identify Clinically Relevant Pulmonary Carcinoid Groups and Unveil the Supra-Carcinoids. Nat Commun (2019) 10(1):3407. 10.1038/s41467-019-11276-9 31431620 PMC6702229

[58] Shen Y Hu F Li C Xu J Zhong R Zhang X. Clinical Features and Outcomes Analysis of Surgical Resected Pulmonary Large-Cell Neuroendocrine Carcinoma With Adjuvant Chemotherapy. Front Oncol (2020) 10:556194. 10.3389/fonc.2020.556194 33335851 PMC7736707

[59] Zheng Q Zheng M Jin Y Shen X Shan L Shen L. ALK-Rearrangement Neuroendocrine Carcinoma of the Lung: A Comprehensive Study of a Rare Case Series and Review of Literature. Onco Targets Ther (2018) 11:4991–8. 10.2147/OTT.S172124 PMC6103612 30154667

[60] Tashiro T Imamura K Tomita Y Tamanoi D Takaki A Sugahara K. Heterogeneous Tumor-Immune Microenvironments Between Primary and Metastatic Tumors in a Patient With ALK Rearrangement-Positive Large Cell Neuroendocrine Carcinoma. Int J Mol Sci (2020) 21(24). 10.3390/ijms21249705 PMC7767140 33352665

[61] Zhou F Hou L Ding T Song Q Chen X Su C. Distinct Clinicopathologic Features, Genomic Characteristics and Survival of Central and Peripheral Pulmonary Large Cell Neuroendocrine Carcinoma: From Different Origin Cells? Lung Cancer (2018) 116:30–7. 10.1016/j.lungcan.2017.12.009 29413048

[62] De Pas TM Giovannini M Manzotti M Trifirò G Toffalorio F Catania C. Large-Cell Neuroendocrine Carcinoma of the Lung Harboring EGFR Mutation and Responding to Gefitinib. J Clin Oncol (2011) 29(34):e819–22. 10.1200/JCO.2011.36.2251 22042963

[63] Tanaka H Hijioka S Hosoda W Ueno M Kobayashi N Ikeda M. Pancreatic Neuroendocrine Carcinoma G3 may be Heterogeneous and Could be Classified Into Two Distinct Groups. Pancreatology (2020). 10.1016/j.pan.2020.07.400 32891532

[64] Yachida S Vakiani E White CM Zhong Y Saunders T Morgan R. Small Cell and Large Cell Neuroendocrine Carcinomas of the Pancreas Are Genetically Similar and Distinct From Well-Differentiated Pancreatic Neuroendocrine Tumors. Am J Surg Pathol (2012) 36(2):173–84. 10.1097/PAS.0b013e3182417d36 PMC3261427 22251937

[65] Fraune C Simon R Hube-Magg C Makrypidi-Fraune G Kluth M Büscheck F. Homogeneous MMR Deficiency Throughout the Entire Tumor Mass Occurs in a Subset of Colorectal Neuroendocrine Carcinomas. Endocr Pathol (2020) 31(2):182–9. 10.1007/s12022-020-09612-7 PMC7250944 32144630

[66] Roberts JA Gonzalez RS Das S Berlin J Shi C. Expression of PD-1 and PD-L1 in Poorly Differentiated Neuroendocrine Carcinomas of the Digestive System: A Potential Target for Anti-PD-1/PD-L1 Therapy. Hum Pathol (2017) 70:49–54. 10.1016/j.humpath.2017.10.003 29037958 PMC5745271

[67] Xing J Ying H Li J Gao Y Sun Z Li J. Immune Checkpoint Markers in Neuroendocrine Carcinoma of the Digestive System. Front Oncol (2020) 10:132. 10.3389/fonc.2020.00132 32181153 PMC7059119

[68] Yang M-W Fu X-L Jiang Y-S Chen X-J Tao L-Y Yang J-Y. Clinical Significance of Programmed Death 1/Programmed Death Ligand 1 Pathway in Gastric Neuroendocrine Carcinomas. World J Gastroenterol (2019) 25(14):1684–96. 10.3748/wjg.v25.i14.1684 PMC6465942 31011254

[69] Pelosi G Bianchi F Dama E Metovic J Barella M Sonzogni A. A Subset of Large Cell Neuroendocrine Carcinomas in the Gastroenteropancreatic Tract May Evolve From Pre-Existing Well-Differentiated Neuroendocrine Tumors. Endocr Pathol (2021). 10.1007/s12022-020-09659-6 33433886

[70] Inage T Nakajima T Fujiwara T Sakairi Y Wada H Suzuki H. Pathological Diagnosis of Pulmonary Large Cell Neuroendocrine Carcinoma by Endobronchial Ultrasound-Guided Transbronchial Needle Aspiration. Thorac Cancer (2018) 9(2):273–7. 10.1111/1759-7714.12576 PMC5792718 29271588

[71] Metro G Ricciuti B Chiari R Baretti M Falcinelli L Giannarelli D. Survival Outcomes and Incidence of Brain Recurrence in High-Grade Neuroendocrine Carcinomas of the Lung: Implications for Clinical Practice. Lung Cancer (2016) 95:82–7. 10.1016/j.lungcan.2016.03.006 27040856

[72] Nielsen K Binderup T Langer SW Kjaer A Knigge P Grøndahl V. P53, Somatostatin Receptor 2a and Chromogranin A Immunostaining as Prognostic Markers in High Grade Gastroenteropancreatic Neuroendocrine Neoplasms. BMC Cancer (2020) 20(1):27. 10.1186/s12885-019-6498-z 31924180 PMC6953213

[73] Righi L Volante M Tavaglione V Billè A Daniele L Angusti T. Somatostatin Receptor Tissue Distribution in Lung Neuroendocrine Tumours: A Clinicopathologic and Immunohistochemical Study of 218 “Clinically Aggressive” Cases. Ann Oncol (2010) 21(3):548–55. 10.1093/annonc/mdp334 19759190

[74] Korse CM Taal BG Vincent A van Velthuysen M-LF Baas P Buning-Kager JCGM. Choice of Tumour Markers in Patients With Neuroendocrine Tumours Is Dependent on the Histological Grade. A Marker Study of Chromogranin A, Neuron Specific Enolase, Progastrin-Releasing Peptide and Cytokeratin Fragments. Eur J Cancer (2012) 48(5):662–71. 10.1016/j.ejca.2011.08.012 21945100

[75] Tian Z Liang C Zhang Z Wen H Feng H Ma Q. Prognostic Value of Neuron-Specific Enolase for Small Cell Lung Cancer: A Systematic Review and Meta-Analysis. World J Surg Oncol (2020) 18(1):116. 10.1186/s12957-020-01894-9 32473655 PMC7261386

[76] Walter T Tougeron D Baudin E Le Malicot K Lecomte T Malka D. Poorly Differentiated Gastro-Entero-Pancreatic Neuroendocrine Carcinomas: Are They Really Heterogeneous? Insights From the FFCD-GTE National Cohort. Eur J Cancer (2017) 79:158–65. 10.1016/j.ejca.2017.04.009 28501762

[77] Fournel L Falcoz PE Alifano M Charpentier M-C Boudaya M-S Magdeleinat P. Surgical Management of Pulmonary Large Cell Neuroendocrine Carcinomas: A 10-Year Experience. Eur J Cardiothorac Surg (2013) 43(1):111–4. 10.1093/ejcts/ezs174 22529187

[78] Zacharias J Nicholson AG Ladas GP Goldstraw P. Large Cell Neuroendocrine Carcinoma and Large Cell Carcinomas With Neuroendocrine Morphology of the Lung: Prognosis After Complete Resection and Systematic Nodal Dissection. Ann Thorac Surg (2003) 75(2):348–52. 10.1016/s0003-4975(02)04118-8 12607637

[79] Lutfi W Schuchert MJ Dhupar R Sarkaria I Christie NA Yang C-FJ. Sublobar Resection is Associated With Decreased Survival for Patients With Early Stage Large-Cell Neuroendocrine Carcinoma of the Lung. Interact Cardiovasc Thorac Surg (2019) 29(4):517–24. 10.1093/icvts/ivz140 31177277

[80] Kim KW Kim HK Kim J Shim YM Ahn M-J Choi Y-L. Outcomes of Curative-Intent Surgery and Adjuvant Treatment for Pulmonary Large Cell Neuroendocrine Carcinoma. World J Surg (2017) 41(7):1820–7. 10.1007/s00268-017-3908-8 28204910

[81] Mosquera C Koutlas NJ Fitzgerald TL. Localized High-Grade Gastroenteropancreatic Neuroendocrine Tumors: Defining Prognostic and Therapeutic Factors for a Disease of Increasing Clinical Significance. Eur J Surg Oncol (2016) 42(10):1471–7. 10.1016/j.ejso.2016.07.137 27528467

[82] Haugvik S-P Janson ET Österlund P Langer SW Falk RS Labori KJ. Surgical Treatment as a Principle for Patients With High-Grade Pancreatic Neuroendocrine Carcinoma: A Nordic Multicenter Comparative Study. Ann Surg Oncol (2016) 23(5):1721–8. 10.1245/s10434-015-5013-2 26678407

[83] Iyoda A Hiroshima K Moriya Y Takiguchi Y Sekine Y Shibuya K. Prospective Study of Adjuvant Chemotherapy for Pulmonary Large Cell Neuroendocrine Carcinoma. Ann Thorac Surg (2006) 82(5):1802–7. 10.1016/j.athoracsur.2006.05.109 17062251

[84] Kenmotsu H Niho S Ito T Ishikawa Y Noguchi M Tada H. A Pilot Study of Adjuvant Chemotherapy With Irinotecan and Cisplatin for Completely Resected High-Grade Pulmonary Neuroendocrine Carcinoma (Large Cell Neuroendocrine Carcinoma and Small Cell Lung Cancer). Lung Cancer (2014) 84(3):254–8. 10.1016/j.lungcan.2014.03.007 24679951

[85] Kenmotsu H Niho S Tsuboi M Wakabayashi M Ishii G Nakagawa K. Randomized Phase III Study of Irinotecan Plus Cisplatin Versus Etoposide Plus Cisplatin for Completely Resected High-Grade Neuroendocrine Carcinoma of the Lung: Jcog1205/1206. J Clin Oncol (2020) 38(36):4292–301. 10.1200/JCO.20.01806 33136471

[86] Rossi G Cavazza A Marchioni A Longo L Migaldi M Sartori G. Role of Chemotherapy and the Receptor Tyrosine Kinases KIT, PDGFRalpha, PDGFRbeta, and Met in Large-Cell Neuroendocrine Carcinoma of the Lung. J Clin Oncol (2005) 23(34):8774–85. 10.1200/JCO.2005.02.8233 16314638

[87] Veronesi G Morandi U Alloisio M Terzi A Cardillo G Filosso P. Large Cell Neuroendocrine Carcinoma of the Lung: A Retrospective Analysis of 144 Surgical Cases. Lung Cancer (2006) 53(1):111–5. 10.1016/j.lungcan.2006.03.007 16697073

[88] Iyoda A Hiroshima K Moriya Y Iwadate Y Takiguchi Y Uno T. Postoperative Recurrence and the Role of Adjuvant Chemotherapy in Patients With Pulmonary Large-Cell Neuroendocrine Carcinoma. J Thorac Cardiovasc Surg (2009) 138(2):446–53. 10.1016/j.jtcvs.2008.12.037 19619794

[89] Sarkaria IS Iyoda A Roh MS Sica G Kuk D Sima CS. Neoadjuvant and Adjuvant Chemotherapy in Resected Pulmonary Large Cell Neuroendocrine Carcinomas: A Single Institution Experience. Ann Thorac Surg (2011) 92(4):1180–6; discussion 1186. 10.1016/j.athoracsur.2011.05.027 21867986

[90] Saji H Tsuboi M Matsubayashi J Miyajima K Shimada Y Imai K. Clinical Response of Large Cell Neuroendocrine Carcinoma of the Lung to Perioperative Adjuvant Chemotherapy. Anticancer Drugs (2010) 21(1):89–93. 10.1097/CAD.0b013e328330fd79 19770636

[91] Kujtan L Muthukumar V Kennedy KF Davis JR Masood A Subramanian J. The Role of Systemic Therapy in the Management of Stage I Large Cell Neuroendocrine Carcinoma of the Lung. J Thorac Oncol (2018) 13(5):707–14. 10.1016/j.jtho.2018.01.019 29391287

[92] Raman V Jawitz OK Yang C-FJ Tong BC D’Amico TA Berry MF. Adjuvant Therapy for Patients With Early Large Cell Lung Neuroendocrine Cancer: A National Analysis. Ann Thorac Surg (2019) 108(2):377–83. 10.1016/j.athoracsur.2019.03.053 PMC7282501 31004586

[93] Eba J Kenmotsu H Tsuboi M Niho S Katayama H Shibata T. A Phase III Trial Comparing Irinotecan and Cisplatin With Etoposide and Cisplatin in Adjuvant Chemotherapy for Completely Resected Pulmonary High-Grade Neuroendocrine Carcinoma (JCOG1205/1206). Jpn J Clin Oncol (2014) 44(4):379–82. 10.1093/jjco/hyt233 24474818

[94] Smith JD Reidy DL Goodman KA Shia J Nash GM. A Retrospective Review of 126 High-Grade Neuroendocrine Carcinomas of the Colon and Rectum. Ann Surg Oncol (2014) 21(9):2956–62. 10.1245/s10434-014-3725-3 PMC4521622 24763982

[95] Haugvik S-P Kaemmerer D Gaujoux S Labori KJ Verbeke CS Gladhaug IP. Pathology and Surgical Treatment of High-Grade Pancreatic Neuroendocrine Carcinoma: An Evolving Landscape. Curr Oncol Rep (2016) 18(5):28. 10.1007/s11912-016-0518-9 26984415

[96] Liu D-J Fu X-L Liu W Zheng L-Y Zhang J-F Huo Y-M. Clinicopathological, Treatment, and Prognosis Study of 43 Gastric Neuroendocrine Carcinomas. World J Gastroenterol (2017) 23(3):516–24. 10.3748/wjg.v23.i3.516 PMC5291857 28210088

[97] Jiang Y Lei C Zhang X Cui Y Che K Shen H. Double-Edged Role of Radiotherapy in Patients With Pulmonary Large-Cell Neuroendocrine Carcinoma. J Cancer (2019) 10(25):6422–30. 10.7150/jca.32446 PMC6856741 31772675

[98] Gu J Gong D Wang Y Chi B Zhang J Hu S. The Demographic and Treatment Options for Patients With Large Cell Neuroendocrine Carcinoma of the Lung. Cancer Med (2019) 8(6):2979–93. 10.1002/cam4.2188 PMC6558599 31087628

[99] Wegner RE Abel S Colonias A. Stereotactic Ablative Body Radiotherapy Versus Conventionally Fractionated Radiotherapy for Early Stage Large Cell Neuroendocrine Carcinoma of the Lung. Lung Cancer Manage (2020) 9(3):LMT32. 10.2217/lmt-2020-0004 PMC7399604 32774465

[100] Rieber J Schmitt J Warth A Muley T Kappes J Eichhorn F. Outcome and Prognostic Factors of Multimodal Therapy for Pulmonary Large-Cell Neuroendocrine Carcinomas. Eur J Med Res (2015) 20:64. 10.1186/s40001-015-0158-9 26272455 PMC4536693

[101] Prelaj A Rebuzzi SE Del Bene G Giròn Berrìos JR Emiliani A De Filippis L. Evaluation of the Efficacy of Cisplatin-Etoposide and the Role of Thoracic Radiotherapy and Prophylactic Cranial Irradiation in LCNEC. ERJ Open Res (2017) 3(1). 10.1183/23120541.00128-2016 PMC5370316 28382303

[102] Wegner RE Hasan S Williamson RW Finley G Fuhrer R Colonias A. Management of Brain Metastases From Large Cell Neuroendocrine Carcinoma of the Lung: Improved Outcomes With Radiosurgery. Acta Oncol (2019) 58(4):499–504. 10.1080/0284186X.2018.1564841 30732516

[103] Le Treut J Sault MC Lena H Souquet PJ Vergnenegre A Le Caer H. Multicentre Phase II Study of Cisplatin-Etoposide Chemotherapy for Advanced Large-Cell Neuroendocrine Lung Carcinoma: The GFPC 0302 Study. Ann Oncol (2013) 24(6):1548–52. 10.1093/annonc/mdt009 23406729

[104] Derks JL van Suylen RJ Thunnissen E den Bakker MA Groen HJ Smit EF. Chemotherapy for Pulmonary Large Cell Neuroendocrine Carcinomas: Does the Regimen Matter? Eur Respir J (2017) 49(6). 10.1183/13993003.01838-2016 PMC5898951 28572122

[105] Derks JL Leblay N Thunnissen E van Suylen RJ den Bakker M Groen HJM. Molecular Subtypes of Pulmonary Large-Cell Neuroendocrine Carcinoma Predict Chemotherapy Treatment Outcome. Clin Cancer Res (2018) 24(1):33–42. 10.1158/1078-0432.CCR-17-1921 29066508

[106] Zhuo M Guan Y Yang X Hong L Wang Y Li Z. The Prognostic and Therapeutic Role of Genomic Subtyping by Sequencing Tumor or Cell-Free DNA in Pulmonary Large-Cell Neuroendocrine Carcinoma. Clin Cancer Res (2020) 26(4):892–901. 10.1158/1078-0432.CCR-19-0556 31694833 PMC7024651

[107] Li J Lu M Lu Z Li Z Liu Y Yang L. Irinotecan Plus Cisplatin Followed by Octreotide Long-Acting Release Maintenance Treatment in Advanced Gastroenteropancreatic Neuroendocrine Carcinoma: IPO-NEC Study. Oncotarget (2017) 8(15):25669–78. 10.18632/oncotarget.12900 PMC5421960 27788498

[108] Alifieris CE Griniatsos J Delis SG Nikolaou M Avgoustou C Panagiotidis MI. Capecitabine, Oxaliplatin, Irinotecan, and Bevacizumab Combination Followed by Pazopanib Plus Capecitabine Maintenance for High-Grade Gastrointestinal Neuroendocrine Carcinomas. Am J Clin Oncol (2020) 43(5):305–10. 10.1097/COC.0000000000000668 32343515

[109] Mitry E Baudin E Ducreux M Sabourin JC Rufié P Aparicio T. Treatment of Poorly Differentiated Neuroendocrine Tumours With Etoposide and Cisplatin. Br J Cancer (1999) 81(8):1351–5. 10.1038/sj.bjc.6690325 PMC2362979 10604732

[110] Terashima T Morizane C Hiraoka N Tsuda H Tamura T Shimada Y. Comparison of Chemotherapeutic Treatment Outcomes of Advanced Extrapulmonary Neuroendocrine Carcinomas and Advanced Small-Cell Lung Carcinoma. Neuroendocrinology (2012) 96(4):324–32. 10.1159/000338794 22572060

[111] Du Z Wang Y Zhou Y Wen F Li Q. First-Line Irinotecan Combined With 5-Fluorouracil and Leucovorin for High-Grade Metastatic Gastrointestinal Neuroendocrine Carcinoma. Tumori (2013) 99(1):57–60. 10.1700/1248.13789 23549001

[112] Yamaguchi T Machida N Morizane C Kasuga A Takahashi H Sudo K. Multicenter Retrospective Analysis of Systemic Chemotherapy for Advanced Neuroendocrine Carcinoma of the Digestive System. Cancer Sci (2014) 105(9):1176–81. 10.1111/cas.12473 PMC4462387 24975505

[113] Bongiovanni A Riva N Ricci M Liverani C La Manna F De Vita A. First-Line Chemotherapy in Patients With Metastatic Gastroenteropancreatic Neuroendocrine Carcinoma. Onco Targets Ther (2015) 8:3613–9. 10.2147/OTT.S91971 PMC4671803 26664145

[114] Kasahara N Wakuda K Omori S Nakashima K Ono A Taira T. Amrubicin Monotherapy may be an Effective Second-Line Treatment for Patients With Large-Cell Neuroendocrine Carcinoma or High-Grade non-Small-Cell Neuroendocrine Carcinoma. Mol Clin Oncol (2017) 6(5):718–22. 10.3892/mco.2017.1198 PMC5431530 28529747

[115] Yoshida H Sekine I Tsuta K Horinouchi H Nokihara H Yamamoto N. Amrubicin Monotherapy for Patients With Previously Treated Advanced Large-Cell Neuroendocrine Carcinoma of the Lung. JPN J Clin Oncol (2011) 41(7):897–901. 10.1093/jjco/hyr065 21636606

[116] Shimada Y Niho S Ishii G Hishida T Yoshida J Nishimura M. Clinical Features of Unresectable High-Grade Lung Neuroendocrine Carcinoma Diagnosed Using Biopsy Specimens. Lung Cancer (2012) 75(3):368–73. 10.1016/j.lungcan.2011.08.012 21920624

[117] Bongiovanni A Liverani C Pusceddu S Leo S Di Meglio G Tamberi S. Randomised Phase II Trial of CAPTEM or FOLFIRI as SEcond-Line Therapy in NEuroendocrine CArcinomas and Exploratory Analysis of Predictive Role of PET/CT Imaging and Biological Markers (SENECA Trial): A Study Protocol. BMJ Open (2020) 10(7):e034393. 10.1136/bmjopen-2019-034393 PMC7371236 32690499

[118] Ando T Hosokawa A Yoshita H Ueda A Kajiura S Mihara H. Amrubicin Monotherapy for Patients With Platinum-Refractory Gastroenteropancreatic Neuroendocrine Carcinoma. Gastroenterol Res Pract (2015) 2015:425876. 10.1155/2015/425876 26199623 PMC4493294

[119] Hadoux J Malka D Planchard D Scoazec JY Caramella C Guigay J. Post-First-Line FOLFOX Chemotherapy for Grade 3 Neuroendocrine Carcinoma. Endocr Relat Cancer (2015) 22(3):289–98. 10.1530/ERC-15-0075 25770151

[120] Nio K Arita S Isobe T Kusaba H Kohashi K Kajitani T. Amrubicin Monotherapy for Patients With Extrapulmonary Neuroendocrine Carcinoma After Platinum-Based Chemotherapy. Cancer Chemother Pharmacol (2015) 75(4):829–35. 10.1007/s00280-015-2706-y 25702050

[121] McNamara MG Frizziero M Jacobs T Lamarca A Hubner RA Valle JW. Second-Line Treatment in Patients With Advanced Extra-Pulmonary Poorly Differentiated Neuroendocrine Carcinoma: A Systematic Review and Meta-Analysis. Ther Adv Med Oncol (2020) 12:1758835920915299. 10.1177/1758835920915299 32426044 PMC7222242

[122] Horn L Mansfield AS Szczęsna A Havel L Krzakowski M Hochmair MJ. First-Line Atezolizumab Plus Chemotherapy in Extensive-Stage Small-Cell Lung Cancer. N Engl J Med (2018) 379(23):2220–9. 10.1056/NEJMoa1809064 30280641

[123] Levra MG Mazieres J Valette CA Molinier O Planchard D Frappat V. P1.07-012 Efficacy of Immune Checkpoint Inhibitors in Large Cell Neuroendocrine Lung Cancer: Results From a French Retrospective Cohort. J Thorac Oncol (2017) 12(1):S702–3. 10.1016/j.jtho.2016.11.923

[124] Oda R Okuda K Yamashita Y Sakane T Tatematsu T Yokota K. Long-Term Survivor of Pulmonary Combined Large Cell Neuroendocrine Carcinoma Treated With Nivolumab. Thorac Cancer (2020) 11(7):2036–9. 10.1111/1759-7714.13471 PMC7327674 32379390

[125] Takimoto Sato M Ikezawa Y Sato M Suzuki A Kawai Y. Large Cell Neuroendocrine Carcinoma of the Lung That Responded to Nivolumab: A Case Report. Mol Clin Oncol (2020) 13(1):43–7. 10.3892/mco.2020.2045 PMC7265227 32499913

[126] Sherman S Rotem O Shochat T Zer A Moore A Dudnik E. Efficacy of Immune Check-Point Inhibitors (ICPi) in Large Cell Neuroendocrine Tumors of Lung (LCNEC). Lung Cancer (2020) 143:40–6. 10.1016/j.lungcan.2020.03.008 32203769

[127] Patel SP Othus M Chae YK Giles FJ Hansel DE Singh PP. A Phase II Basket Trial of Dual Anti-CTLA-4 and Anti-PD-1 Blockade in Rare Tumors (DART SWOG 1609) in Patients With Nonpancreatic Neuroendocrine Tumors. Clin Cancer Res (2020) 26(10):2290–6. 10.1158/1078-0432.CCR-19-3356 PMC7231627 31969335

[128] Shirasawa M Yoshida T Takayanagi D Shiraishi K Yagishita S Sekine K. Activity and Immune Correlates of Programmed Deaht-1 Blockade Therapy in Patients With Advanced Large Cell Neuroendocrine Carcinoma. Clin Lung Cancer (2021). 10.1016/j.cllc.2021.02.003 33722498

[129] Cescon DW Bratman SV Chan SM Siu LL. Circulating Tumor DNA and Liquid Biopsy in Oncology. Nat Cancer (2020) 1(3):276–90. 10.1038/s43018-020-0043-5 35122035

[130] Christopoulos P Schneider MA Bozorgmehr F Kuon J Engel-Riedel W Kollmeier J. Large Cell Neuroendocrine Lung Carcinoma Induces Peripheral T-Cell Repertoire Alterations With Predictive and Prognostic Significance. Lung Cancer (2018) 119:48–55. 10.1016/j.lungcan.2018.03.002 29656752

[131] Ready NE Ott PA Hellmann MD Zugazagoitia J Hann CL de Braud F. Nivolumab Monotherapy and Nivolumab Plus Ipilimumab in Recurrent Small Cell Lung Cancer: Results From the Checkmate 032 Randomized Cohort. J Thorac Oncol (2020) 15(3):426–35. 10.1016/j.jtho.2019.10.004 31629915

[132] Marabelle A Fakih M Lopez J Shah M Shapira-Frommer R Nakagawa K. Association of Tumour Mutational Burden With Outcomes in Patients With Advanced Solid Tumours Treated With Pembrolizumab: Prospective Biomarker Analysis of the Multicohort, Open-Label, Phase 2 KEYNOTE-158 Study. Lancet Oncol (2020) 21(10):1353–65. 10.1016/S1470-2045(20)30445-9 32919526

[133] Samstein RM Lee C-H Shoushtari AN Hellmann MD Shen R Janjigian YY. Tumor Mutational Load Predicts Survival After Immunotherapy Across Multiple Cancer Types. Nat Genet (2019) 51(2):202–6. 10.1038/s41588-018-0312-8 PMC6365097 30643254

[134] Shao C Li G Huang L Pruitt S Castellanos E Frampton G. Prevalence of High Tumor Mutational Burden and Association With Survival in Patients With Less Common Solid Tumors. JAMA Netw Open (2020) 3(10):e2025109. 10.1001/jamanetworkopen.2020.25109 33119110 PMC7596577

[135] Sabari JK Lok BH Laird JH Poirier JT Rudin CM. Unravelling the Biology of SCLC: Implications for Therapy. Nat Rev Clin Oncol (2017) 14(9):549–61. 10.1038/nrclinonc.2017.71 PMC5843484 28534531

[136] Kim HS Han J-Y Lee GK Lee JH. PARP1 Expression in High-Grade Neuroendocrine Carcinoma of the Lung. JCO (2018) 36(15_suppl):e20562–2. 10.1200/JCO.2018.36.15_suppl.e20562

[137] Bondgaard A-LRØ Poulsen TT Poulsen HS Skov BG. Different Expression of EZH2, BMI1 and Ki67 in Low and High Grade Neuroendocrine Tumors of the Lung. Cancer Biomark (2012) 11(2-3):123–8. 10.3233/CBM-2012-0269 PMC13016217 23011159

[138] Deng S-M Yan X-C Liang L Wang L Liu Y Duan J-L. The Notch Ligand Delta-Like 3 Promotes Tumor Growth and Inhibits Notch Signaling in Lung Cancer Cells in Mice. Biochem Biophys Res Commun (2017) 483(1):488–94. 10.1016/j.bbrc.2016.12.117 28007595

[139] Hermans BCM Derks JL Thunnissen E van Suylen RJ den Bakker MA Groen HJM. DLL3 Expression in Large Cell Neuroendocrine Carcinoma (LCNEC) and Association With Molecular Subtypes and Neuroendocrine Profile. Lung Cancer (2019) 138:102–8. 10.1016/j.lungcan.2019.10.010 31678831

[140] Matsuo K Taniguchi K Hamamoto H Ito Y Futaki S Inomata Y. Delta-Like 3 Localizes to Neuroendocrine Cells and Plays a Pivotal Role in Gastrointestinal Neuroendocrine Malignancy. Cancer Sci (2019). 10.1111/cas.14157 PMC6778628 31369178

[141] Rudin CM Pietanza MC Bauer TM Ready N Morgensztern D Glisson BS. Rovalpituzumab Tesirine, a DLL3-Targeted Antibody-Drug Conjugate, in Recurrent Small-Cell Lung Cancer: A First-in-Human, First-in-Class, Open-Label, Phase 1 Study. Lancet Oncol (2017) 18(1):42–51. 10.1016/S1470-2045(16)30565-4 27932068 PMC5481162

[142] Ogawa H Sakai Y Nishio W Fujibayashi Y Nishikubo M Nishioka Y. DLL3 Expression Is a Predictive Marker of Sensitivity to Adjuvant Chemotherapy for Pulmonary LCNEC. Thorac Cancer (2020) 11(9):2561–9. 10.1111/1759-7714.13574 PMC7471044 32691982

[143] Morgensztern D Besse B Greillier L Santana-Davila R Ready N Hann CL. Efficacy and Safety of Rovalpituzumab Tesirine in Third-Line and Beyond Patients With DLL3-Expressing, Relapsed/Refractory Small-Cell Lung Cancer: Results From the Phase II TRINITY Study. Clin Cancer Res (2019) 25(23):6958–66. 10.1158/1078-0432.CCR-19-1133 PMC7105795 31506387

[144] Morgensztern D Johnson M Rudin CM Rossi M Lazarov M Brickman D. SC-002 in Patients With Relapsed or Refractory Small Cell Lung Cancer and Large Cell Neuroendocrine Carcinoma: Phase 1 Study. Lung Cancer (2020) 145:126–31. 10.1016/j.lungcan.2020.04.017 PMC8173700 32438272

[145] Owen DH Giffin MJ Bailis JM Smit M-AD Carbone DP He K. DLL3: An Emerging Target in Small Cell Lung Cancer. J Hematol Oncol (2019) 12(1):61. 10.1186/s13045-019-0745-2 31215500 PMC6582566

[146] Miyagawa-Hayashino A Okada S Takeda-Miyata N Takashima Y Yamada T Takemura Y. TTF-1 and C-MYC-Defined Phenotypes of Large Cell Neuroendocrine Carcinoma and Delta-Like Protein 3 Expression for Treatment Selection. Appl Immunohistochem Mol Morphol (2020). 10.1097/PAI.0000000000000875 PMC8132912 33031101

[147] Hook KE Garza SJ Lira ME Ching KA Lee NV Cao J. An Integrated Genomic Approach to Identify Predictive Biomarkers of Response to the Aurora Kinase Inhibitor PF-03814735. Mol Cancer Ther (2012) 11(3):710–9. 10.1158/1535-7163.MCT-11-0184 22222631

[148] Escala Cornejo RA García-Talavera P Navarro Martin M Pérez López B García Muñoz M Tamayo Alonso MP. Large Cell Neuroendocrine Carcinoma of the Lung With Atypical Evolution and a Remarkable Response to Lutetium Lu 177 Dotatate. Ann Nucl Med (2018) 32(8):568–72. 10.1007/s12149-018-1276-6 30051167

[149] Nocuń A Chrapko B Gołębiewska R Stefaniak B Czekajska-Chehab E. Evaluation of Somatostatin Receptors in Large Cell Pulmonary Neuroendocrine Carcinoma With 99mtc-EDDA/HYNIC-TOC Scintigraphy. Nucl Med Commun (2011) 32(6):522–9. 10.1097/MNM.0b013e32834508b3 21383640

[150] Makino T Mikami T Hata Y Otsuka H Koezuka S Isobe K. Comprehensive Biomarkers for Personalized Treatment in Pulmonary Large Cell Neuroendocrine Carcinoma: A Comparative Analysis With Adenocarcinoma. Ann Thorac Surg (2016) 102(5):1694–701. 10.1016/j.athoracsur.2016.04.100 27368130

[151] Filosso PL Ruffini E Oliaro A Rena O Casadio C Mancuso M. Large-Cell Neuroendocrine Carcinoma of the Lung: A Clinicopathologic Study of Eighteen Cases and the Efficacy of Adjuvant Treatment With Octreotide. J Thorac Cardiovasc Surg (2005) 129(4):819–24. 10.1016/j.jtcvs.2004.05.023 15821649

